# Hemodynamic response after preoperative embolization in thymoma-associated myasthenia gravis: associations with necrosis, HIF-1α expression, and CD163-positive macrophages

**DOI:** 10.3389/fimmu.2026.1926398

**Published:** 2026-08-18

**Authors:** Hao Yang, Xingtao Pi, Wendong Cao, Duqiang Li

**Affiliations:** Shanxi Bethune Hospital, Shanxi Academy of Medical Sciences, Third Hospital of Shanxi Medical University, Tongji Shanxi Hospital, Taiyuan, Shanxi, China

**Keywords:** CD163-positive macrophages, hemodynamic response, HIF-1α, immune microenvironment, ischemic necrosis, preoperative embolization, thymoma-associated myasthenia gravis

## Abstract

**Background:**

Thymoma-associated myasthenia gravis is a distinct clinicopathological setting in which thymic epithelial tumor biology, autoimmunity, and perioperative neurological risk overlap. In selected hypervascular thymomas, preoperative transarterial embolization is used before surgical resection, but the tissue-level correlates of the angiographic response remain unclear.

**Methods:**

This single-center retrospective translational cohort study included 64 patients with thymoma-associated myasthenia gravis who underwent clinically indicated preoperative embolization followed by thymectomy between January 2018 and June 2025. The primary exposure was angiographic tumor blush reduction measured on digital subtraction angiography. Primary outcomes were tumor necrosis proportion, HIF-1α H-score, and CD163-positive macrophage density. Patients were descriptively stratified into low-, intermediate-, and high-response groups according to tumor blush reduction, while continuous blush reduction was retained for regression analyses.

**Results:**

Among 214 screened patients with thymic epithelial tumors, 64 met the eligibility criteria. Median tumor blush reduction was 56.2%. Tumor necrosis proportion increased across the low-, intermediate-, and high-response groups, with values of 30.6% ± 19.4%, 40.2% ± 23.5%, and 56.1% ± 24.6%, respectively (P = 0.003; FDR q = 0.036). HIF-1α H-score showed a similar pattern (111.4 ± 51.6, 134.8 ± 60.3, and 169.5 ± 61.0; P = 0.008; FDR q = 0.048). CAIX and CD163-positive macrophage density showed unadjusted between-group differences, whereas VEGF-A, CD8-positive T-cell density, FOXP3-positive T-cell density, PD-L1 tumor proportion score, CD8/FOXP3 ratio, and the composite immune-enriched phenotype showed broader overlap across groups. Exploratory CD20 and CXCL13 analyses did not show a graded increase across hemodynamic response strata. In multivariable analyses, each 10% increase in tumor blush reduction was associated with greater necrosis proportion and higher HIF-1α H-score. The association between blush reduction and HIF-1α H-score was attenuated after adjustment for necrosis proportion. Exploratory postoperative MG outcomes did not show a clear graded association with angiographic response.

**Conclusion:**

In embolized thymoma-associated myasthenia gravis, greater angiographic devascularization was associated mainly with ischemic necrosis and HIF-1α expression. Immune findings were heterogeneous, with CD163-positive macrophage density showing an exploratory signal rather than a uniform immune shift. These results support an association-based interpretation and require prospective validation.

## 1 Introduction

Thymic epithelial tumors are uncommon anterior mediastinal malignancies with marked histological and immunological heterogeneity. Although rare in population-based registries, thymomas are among the most frequent epithelial neoplasms of the adult anterior mediastinum (1). The current World Health Organization classification divides thymomas into types A, AB, B1, B2, and B3 according to epithelial morphology and lymphocytic content, and this classification remains clinically relevant because histological subtype is associated with immune landscape, stage distribution, and surgical management (2).

Myasthenia gravis is an autoimmune disorder of neuromuscular transmission, usually characterized by fluctuating fatigable weakness and autoantibodies against the acetylcholine receptor or other postsynaptic membrane proteins (3). Thymoma-associated myasthenia gravis differs from non-thymomatous myasthenia gravis because the neoplastic thymus may sustain abnormal antigen presentation, altered T-cell selection, and breakdown of immune tolerance (4). In this setting, thymectomy is not only an oncological procedure but also part of perioperative autoimmune disease management, where tumor control, operative safety, and neurological stability are closely linked.

A subset of thymomas is hypervascular, with arterial supply from branches of the internal thoracic, pericardiophrenic, bronchial, intercostal, inferior thyroid, or other mediastinal vessels. In selected hypervascular thoracic tumors, preoperative transarterial embolization can be used to reduce tumor vascularity before resection and improve operative control in anatomically difficult regions (5). However, technical completion of embolization does not necessarily indicate the extent of tissue-level change. Whether greater angiographic devascularization corresponds to increased ischemic necrosis, stronger hypoxia-marker expression, or altered immune features in resected thymoma tissue remains insufficiently defined.

HIF-1α is a central regulator of cellular adaptation to hypoxia and can influence angiogenesis, metabolic reprogramming, inflammatory signaling, and tumor-stromal interaction. After arterial embolization, reduced blood flow may create ischemic and hypoxic tissue regions, particularly around necrotic tumor bed. Studies in transarterial embolization or chemoembolization settings have reported post-procedural activation of HIF-1α and VEGF-related pathways, although the timing and magnitude of these signals may vary by tumor type, residual viable tissue, and sampling interval (6). This provides a rationale for evaluating HIF-1α, CAIX, and VEGF-A in embolized thymoma tissue while avoiding the assumption that embolization alone determines their expression.

The immune microenvironment of thymic epithelial tumors is complex. Thymomas may contain immature thymocyte-like lymphocytes, mature T cells, macrophages, B-cell populations, germinal-center-like structures, and immune-checkpoint molecule expression (7). In thymoma-associated myasthenia gravis, this immune architecture is further shaped by autoimmunity and MG-directed therapy. Recent single-cell and spatial studies suggest that MG-associated thymoma contains disease-relevant epithelial, lymphoid, and myeloid niches that may not be captured by conventional histological subtype alone (8). PD-1 and PD-L1 expression have also been reported in thymic epithelial tumors, although their distribution and clinical meaning vary by subtype and study context (9). For this reason, immune assessment in TAMG should not rely only on broad T-cell or checkpoint markers; B-cell and germinal-center-related markers, including CD20 and CXCL13, may provide additional information about autoimmune thymic lesions.

The relationships among embolization-related hemodynamic response, ischemic necrosis, hypoxia-related marker expression, and immune microenvironmental features remain unclear in thymoma-associated myasthenia gravis. This study evaluated a single-center cohort of embolized TAMG patients using paired angiographic, pathological, and immunohistochemical data. The primary objective was to determine whether the degree of angiographic tumor blush reduction was associated with tumor necrosis proportion, HIF-1α expression, and selected immune microenvironment markers in post-embolization thymoma specimens. Exploratory analyses further examined MG-related treatment variables, short-term postoperative MG outcomes, and B-cell/germinal-center markers to clarify whether angiographic response was linked to autoimmune-relevant tissue features.

## 2 Methods

### 2.1 Study design and reporting framework

This single-center retrospective translational cohort study assessed the association between angiographic hemodynamic response to clinically indicated preoperative embolization and subsequent tissue-level pathological, hypoxia-related, and immune microenvironmental features in patients with thymoma-associated myasthenia gravis. Consecutive patients undergoing surgical resection for thymic epithelial tumors between January 2018 and June 2025 were screened for eligibility. This retrospective human cohort study was approved by Shanxi Bethune Hospital (approval No. Sx28174, valid from 2025.10.24 to 2026.10.22). Written informed consent was waived by Shanxi Bethune Hospital because only de-identified retrospective clinical, imaging, procedural, and pathological data were analyzed. The study was performed in compliance with the Declaration of Helsinki and reported in line with the Strengthening the Reporting of Observational Studies in Epidemiology (STROBE) guideline for cohort studies (10).

The analytical cohort was confined to patients with thymoma-associated myasthenia gravis who had undergone clinically indicated preoperative transarterial embolization followed by surgical resection. Patients with sporadic thymoma were not included in the analytical cohort because the study focused on embolized thymoma in the setting of MG-related autoimmune disease, MG-directed therapy, and perioperative neurological outcomes. Non-embolized patients were identified during screening but were not used as a comparator group. The primary exposure was the degree of angiographic tumor devascularization achieved after embolization, rather than embolization status itself. The study therefore evaluated within-cohort associations and was not designed to compare TAMG with sporadic thymoma or embolized with non-embolized thymoma.

### 2.2 Patient selection

Patients were eligible if they satisfied all of the following criteria: age ≥18 years; histologically confirmed thymoma on the resected specimen; clinical diagnosis of thymoma-associated myasthenia gravis; preoperative contrast-enhanced chest CT or CT angiography demonstrating a hypervascular anterior mediastinal tumor; preoperative transarterial embolization before planned resection; availability of both pre-embolization and completion digital subtraction angiography images; surgical resection within 2–5 days after embolization; and adequate formalin-fixed, paraffin-embedded tumor tissue for hematoxylin-eosin evaluation and immunohistochemical staining. Thymoma-associated myasthenia gravis was defined as neurologist-diagnosed MG in a patient with histologically confirmed thymoma, regardless of whether MG symptoms preceded, coincided with, or followed radiological tumor detection.

**Figure 1:**
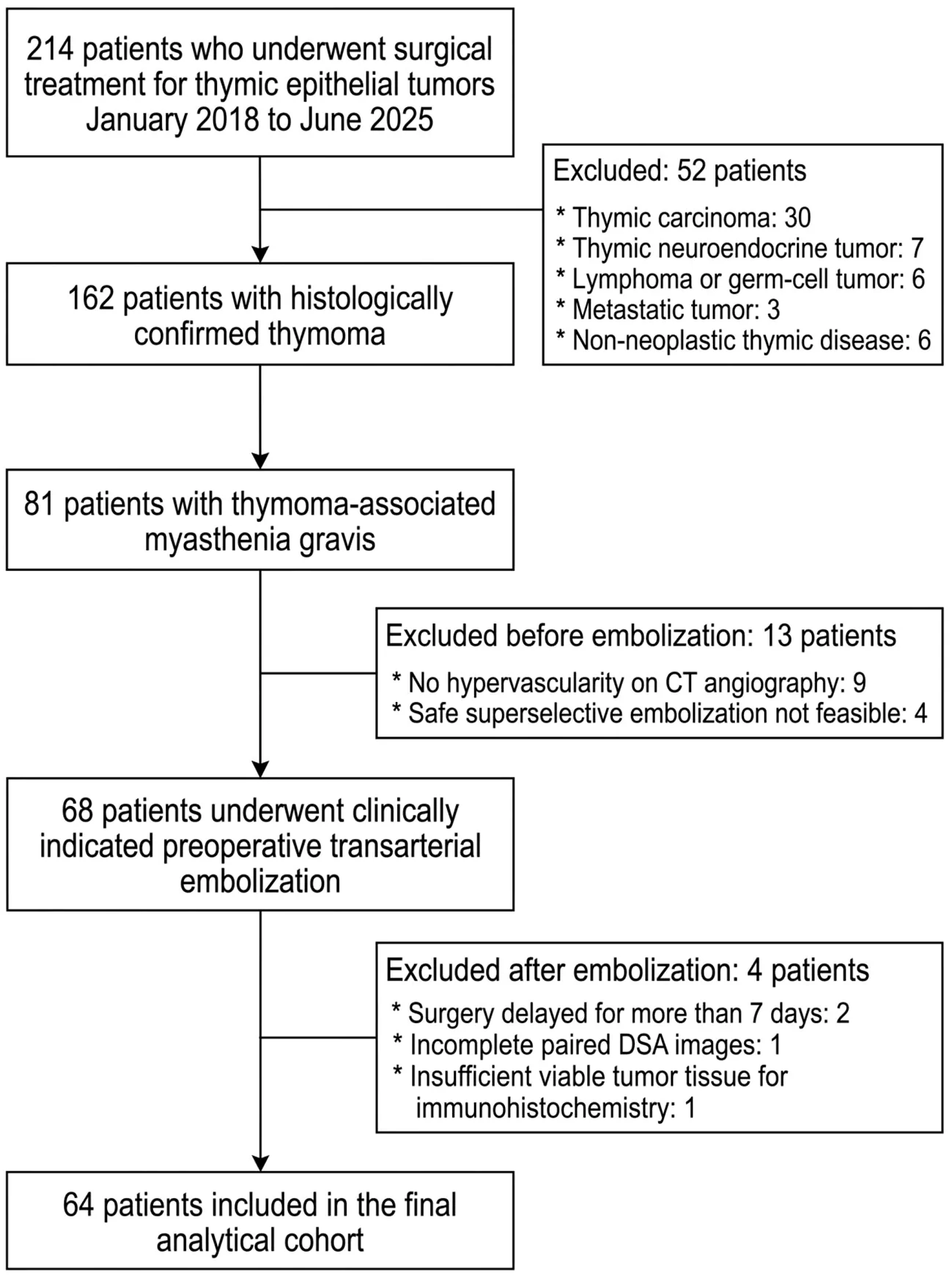
Patient selection flow diagram. Consecutive patients who underwent surgical treatment for thymic epithelial tumors between January 2018 and June 2025 were screened. Patients were excluded according to pathological diagnosis, absence of thymoma-associated myasthenia gravis, including sporadic thymoma without clinical MG, lack of hypervascularity or embolization feasibility, delayed surgery, incomplete angiographic data, or insufficient tumor tissue. The final analytical cohort included 64 patients with embolized thymoma-associated myasthenia gravis.

Exclusion criteria applied if final pathology revealed thymic carcinoma, thymic neuroendocrine tumor, lymphoma, germ-cell tumor, metastatic tumor, or non-neoplastic thymic disease; if the patient had sporadic thymoma without clinical MG; if neoadjuvant chemotherapy, radiotherapy, targeted therapy, or immune checkpoint inhibitor therapy had been administered before embolization; if embolization was performed for emergency bleeding rather than planned preoperative devascularization; if surgery was delayed for more than 7 days after embolization; if the tumor specimen was too fragmented to permit reliable necrosis estimation; or if viable tumor tissue was insufficient for HIF-1α and immune-marker staining. The patient screening process and reasons for exclusion are summarized in **Figure 1**.

The diagnosis of myasthenia gravis was established by neurologists on the basis of fluctuating fatigable weakness, compatible findings on neurological examination, acetylcholine receptor antibody or muscle-specific kinase antibody testing when available, electrophysiological evidence when clinically indicated, and documented response to standard therapeutic interventions. Baseline disease severity was graded according to the Myasthenia Gravis Foundation of America clinical classification and the Quantitative Myasthenia Gravis score assessed closest to the date of embolization (11). When multiple pre-embolization neurological assessments were available, the assessment closest to embolization and before perioperative rescue therapy was used as baseline.

### 2.3 Clinical variables and disease classification

Data extraction from the electronic medical record encompassed baseline, neurological, tumor-related, procedural, and perioperative parameters: age at surgery, sex, smoking history, body mass index, hypertension, diabetes mellitus, coronary artery disease, duration of myasthenia gravis symptoms, MGFA class, QMG score, acetylcholine receptor antibody level, muscle-specific kinase antibody status when available, use of pyridostigmine, corticosteroids, non-corticosteroid immunosuppressants, intravenous immunoglobulin, plasma exchange, tumor maximum diameter, operative approach, completeness of resection, intraoperative blood loss, postoperative myasthenic crisis, intensive-care-unit admission, and postoperative hospital stay. MG-directed therapy was recorded as corticosteroid exposure within 30 days before embolization, prednisone-equivalent dose closest to embolization, non-corticosteroid immunosuppressant use for at least 4 weeks before embolization, intravenous immunoglobulin within 30 days before surgery, plasma exchange within 30 days before surgery, and pyridostigmine use at embolization.

Exploratory MG-related outcomes included postoperative myasthenic crisis during the index hospitalization, respiratory deterioration requiring non-invasive or invasive ventilatory support, postoperative intravenous immunoglobulin or plasma exchange, QMG score at the first postoperative neurological reassessment, QMG score at approximately 3 months after surgery, and acetylcholine receptor antibody level at the closest available postoperative follow-up. These neurological outcomes were analyzed separately from the primary tissue-level endpoints. A comprehensive summary of all prespecified variables—including definitions, units, data sources, and analytical roles—is provided in **Supplementary Table 1**. MG-directed treatment variables and exploratory MG outcome definitions are summarized in **Supplementary Table 2**.

Histological subtyping was performed according to the World Health Organization classification of thymic epithelial tumors, with tumors assigned as type A, AB, B1, B2, or B3 (12). Pathological staging followed the International Thymic Malignancy Interest Group and International Association for the Study of Lung Cancer TNM framework for thymic epithelial neoplasms (13). For multivariable adjustment, the following core covariates were prespecified on clinical grounds: tumor maximum diameter, WHO B2/B3 histology, TNM stage III/IV disease, and embolization-to-surgery interval. Baseline QMG score, corticosteroid exposure, prednisone-equivalent dose, non-corticosteroid immunosuppressant use, intravenous immunoglobulin, and plasma exchange were included in sensitivity analyses for MG severity and autoimmune therapy.

### 2.4 Preoperative imaging protocol and vascular assessment

All patients underwent contrast-enhanced chest CT or CT angiography before embolization. Imaging was acquired on a multidetector CT system using a tube voltage of 100–120 kVp, automatic tube current modulation, collimation of 0.5–1.25 mm, and reconstructed slice thickness of 1.0–1.5 mm. Nonionic iodinated contrast material with a concentration of 350–370 mg iodine/mL was administered via an antecubital vein at a dose of 1.5 mL/kg and an injection rate of 3.0–4.0 mL/s, followed by a 30–40 mL saline flush. Arterial-phase acquisition was triggered by bolus tracking in the descending thoracic aorta at a threshold of 100 HU, whereas venous-phase images were obtained at 65–75 seconds after contrast injection.

Preoperative CT or CT angiography images were independently reviewed by two thoracic radiologists who were blinded to pathological and immunohistochemical findings. Tumor maximum diameter was measured on axial or multiplanar reconstructed views. Tumor enhancement was quantified by placing three non-overlapping regions of interest within enhancing solid tumor components, carefully avoiding macroscopic necrosis, calcification, hemorrhage, and major vessels; the mean value of these three measurements was recorded as the tumor enhancement value. Hypervascularity was defined as visible arterial-phase enhancement exceeding that of adjacent skeletal muscle, accompanied by identifiable tumor-feeding vessels on CT angiography. Feeding-artery anatomy was recorded for procedural planning and descriptive analysis. Any disagreements between the two reviewers were resolved through consensus discussion.

### 2.5 Indication and procedure for preoperative embolization

Preoperative embolization was offered to selected patients with hypervascular thymoma when multidisciplinary consensus between thoracic surgery and interventional radiology teams indicated that devascularization could reduce operative difficulty or enhance the safety of resection. This decision was guided by tumor vascularity, tumor size, proximity to major vessels, suspected local invasion, feeding-artery anatomy, and technical feasibility. The procedural indication was preoperative devascularization of a hypervascular anterior mediastinal tumor; MG-directed medical therapy was managed separately by neurologists. Embolization was withheld when target feeding vessels could not be safely catheterized or when angiography revealed hazardous anastomoses to coronary, spinal, cerebral, or dominant bronchial circulation that posed an unacceptable risk of non-target embolization.

All procedures were conducted by interventional radiologists using a super selective technique. Femoral arterial access served as the default route, with radial access reserved for cases in which femoral access was unsuitable. A 5-French vascular sheath was inserted, followed by selective angiography of potential feeding arteries—including branches of the internal thoracic, pericardiophrenic, bronchial, intercostal, and inferior thyroid arteries, as well as other mediastinal branches when indicated by preoperative CT angiography. Superselective catheterization was achieved using a 2.0–2.7-French microcatheter. Before embolic delivery, angiography was reviewed for feeding-artery anatomy, flow direction, arteriovenous shunting, catheter stability, and hazardous anastomoses.

Polyvinyl alcohol particles constituted the primary embolic agent. Particles of 150–350 μm were employed for distal tumor-bed embolization when no hazardous anastomoses were identified, whereas 350–500 μm particles were chosen when more proximal vascular control was deemed safer. Gelatin sponge particles were added when persistent tumor blush remained after particle embolization and the microcatheter position remained stable. Coils were deployed for large proximal feeders with high-flow shunting or when durable proximal occlusion was required. Embolization was continued until marked reduction of tumor blush and near-stasis in the target feeder were angiographically confirmed. The overall strategy adhered to the established principle that preoperative arterial embolization can reduce vascularity in selected hypervascular thoracic tumors before surgical resection (14).

### 2.6 Quantification of embolization-related hemodynamic response

The primary exposure variable comprised the angiographic hemodynamic response, defined as the percentage reduction in tumor blush between baseline angiography and completion angiography. For each case, pre-embolization and completion DSA frames were selected from the same projection whenever technically feasible. Frames were selected from the phase showing maximal tumor parenchymal blush and were avoided when arterial inflow, venous drainage, respiratory motion, catheter movement, or subtraction artifact obscured the tumor blush. Images were exported in DICOM format and analyzed using ImageJ version 1.54f.

A polygonal region of interest was manually drawn around the visible tumor blush, and a background region of interest was placed over adjacent non-tumor mediastinal soft tissue. The tumor ROI included visible tumor blush while excluding large feeding arteries, proximal embolized vessels, venous pooling, calcification, gross hemorrhage, bony overlap, and major motion or subtraction artifact. The same anatomical tumor-blush boundary was used on completion angiography whenever possible. Background ROIs were placed in the same DSA field over adjacent non-tumor mediastinal soft tissue or chest wall skeletal muscle, avoiding lung fields, cardiac silhouette, large vessels, catheters, guidewires, and artifact. ROI placement was performed blinded to histological necrosis, immunohistochemical scores, and postoperative outcomes.

Background-corrected tumor blush intensity was derived as the mean gray value within the tumor region minus that of the background region. Hemodynamic response was calculated using the following formula: hemodynamic response (%) = [(baseline background-corrected tumor blush intensity − completion background-corrected tumor blush intensity)/baseline background-corrected tumor blush intensity] × 100.

For descriptive graphical purposes, patients were stratified into low-, intermediate-, and high-response groups using prespecified thresholds of less than 45%, 45% to 65%, and greater than 65% tumor blush reduction, respectively. The continuous percentage reduction was retained for all primary regression analyses to avoid information loss from categorical assignment. The response strata were used only for descriptive tables and figures.

### 2.7 Perioperative management and surgical procedure

All patients underwent multidisciplinary preoperative evaluation involving thoracic surgeons, neurologists, anesthesiologists, and interventional radiologists. Medical therapy—including pyridostigmine, corticosteroids, non-corticosteroid immunosuppressants, intravenous immunoglobulin, and plasma exchange—was prescribed according to routine neurological practice and individualized perioperative risk assessment. Patients presenting with bulbar symptoms, respiratory weakness, recent clinical deterioration, or elevated QMG scores received targeted neurological optimization before embolization and surgery. The type and timing of MG-directed therapy were recorded and used in sensitivity analyses because these therapies may influence immune-cell composition and inflammatory marker expression.

Surgical resection was performed within 2–5 days after embolization. The operative procedure comprised extended thymectomy or radical thymoma resection, tailored to tumor extent. The surgical approach—video-assisted thoracoscopic surgery, robotic-assisted thoracic surgery, sternotomy, thoracotomy, or combined approach—was selected based on tumor size, location, suspected invasion, vascular proximity, and operative safety. Completeness of resection was classified as R0, R1, or R2 according to operative and pathological reports. Postoperative neurological monitoring included myasthenic crisis, respiratory deterioration, rescue MG-directed therapy, intensive-care-unit admission, and postoperative QMG reassessment when available.

### 2.8 Tissue processing and assessment of ischemic necrosis

Resected specimens were fixed in 10% neutral buffered formalin for 24–48 hours, sampled in accordance with routine thoracic pathology practice, embedded in paraffin, sectioned at 4 μm, and stained with hematoxylin and eosin. For tumors exhibiting spatial heterogeneity in necrosis, at least one section from the central tumor region, one from the peripheral viable rim, and one from the area closest to the dominant feeding-vessel entry zone were reviewed whenever available. When these regions were represented in separate tissue blocks, all available blocks were reviewed before selecting slides for necrosis quantification and immunohistochemistry.

Tumor necrosis was quantified on digitized hematoxylin-eosin slides. Necrotic areas were operationally defined as regions showing eosinophilic ghost tumor architecture, coagulative necrosis, karyorrhectic debris, or infarct-like necrotic tumor bed within the thymoma tissue. Cystic degeneration, old hemorrhage without definite tumor-cell necrosis, cautery artifact, and non-tumor fibrotic mediastinal tissue were excluded from the numerator. Total tumor area was defined as viable plus necrotic thymoma tissue, excluding surrounding fat, capsule, and non-neoplastic thymic elements. Necrosis proportion was calculated as necrotic tumor area divided by total tumor area and expressed as a percentage. For downstream marker assessment, viable tumor regions were annotated separately from necrotic tumor bed and peri-necrotic viable rim to reduce misclassification of necrotic debris as positive immunostaining.

Two pathologists independently assessed necrosis while blinded to angiographic response, CT findings, embolization details, MG severity, MG-directed therapy, and postoperative outcomes. When the difference between the two estimates exceeded 10 percentage points, the slides were jointly reviewed to achieve a consensus value.

### 2.9 Immunohistochemistry for hypoxia-related markers

Immunohistochemical staining was applied to 4-μm formalin-fixed, paraffin-embedded tumor sections. Following deparaffinization and rehydration, heat-induced antigen retrieval was performed using citrate buffer at pH 6.0 for HIF-1α and VEGF-A, and EDTA buffer at pH 9.0 for CAIX. Endogenous peroxidase activity was blocked with 3% hydrogen peroxide. Sections were then incubated with primary antibodies directed against HIF-1α (clone H1alpha67), CAIX (clone TH22), and VEGF-A (clone VG1). Detection was accomplished using a polymer-based horseradish peroxidase system with 3,3′-diaminobenzidine as chromogen, followed by hematoxylin counterstaining. Placental tissue and known positive tumor tissue served as external positive controls depending on the marker, and negative controls were processed by omitting the primary antibody.

Expression of HIF-1α, CAIX, and VEGF-A was evaluated using the H-score method. Staining intensity was graded as 0, 1, 2, or 3, and the percentage of tumor cells at each intensity level was recorded. The H-score was computed as Σ Pi × i, where Pi represented the percentage of tumor cells stained at each intensity and i denoted the corresponding intensity score, yielding a range from 0 to 300. For HIF-1α, nuclear staining was prioritized, with cytoplasmic staining recorded only when accompanied by definite nuclear positivity. CAIX was scored based on membranous or membranous-cytoplasmic staining, whereas VEGF-A was scored on cytoplasmic staining in viable tumor cells. Scoring was performed in viable tumor regions, with peri-necrotic viable tumor recorded when present and necrotic debris excluded. These markers were selected because post-embolization ischemic and hypoxic tissue conditions may be associated with HIF-1α-mediated hypoxia-adaptation and angiogenic signaling pathways (15).

### 2.10 Immune microenvironment quantification

Quantification of CD8-positive T cells, FOXP3-positive regulatory T cells, CD163-positive macrophages, and PD-L1 expression was performed on 4-μm tumor sections. Antigen retrieval employed citrate buffer at pH 6.0 for CD8 and EDTA buffer at pH 9.0 for FOXP3, CD163, and PD-L1. The following primary antibody clones were utilized: CD8 (clone C8/144B), FOXP3 (clone 236A/E7), CD163 (clone 10D6), and PD-L1 (clone 22C3). Tonsil tissue and known positive tumor tissue served as positive controls according to the marker tested. To address TAMG-associated B-cell and germinal-center pathology, CD20 and CXCL13 were added as exploratory immune markers when sufficient residual formalin-fixed, paraffin-embedded tissue was available. CD20 was used to assess B-cell aggregates, and CXCL13 was used to assess germinal-center-associated chemokine expression in viable tumor and peritumoral thymic tissue.

CD8-positive T cells, FOXP3-positive regulatory T cells, CD163-positive macrophages, CD20-positive B cells, and CXCL13-positive areas were quantified on digitized whole-slide images using QuPath version 0.5.1. Viable tumor regions were manually annotated while excluding necrotic areas, hemorrhage, large vessels, fibrotic capsule, and non-neoplastic thymic tissue. For CD20 and CXCL13, lymphoid aggregates and germinal-center-like foci in adjacent non-neoplastic thymic tissue were recorded separately from intratumoral immune staining when present. Cell detection was performed via hematoxylin nuclear segmentation followed by DAB-positive cell classification. All automated outputs were manually reviewed by a pathologist, and false-positive staining arising from crushed tissue, pigment, edge artifact, or necrotic debris was removed. Marker density was expressed as positive cells per mm² of viable tumor area. CXCL13 was quantified as the percentage of annotated viable tissue area showing definite DAB-positive staining, because its staining pattern was not limited to discrete single cells. This immune panel was chosen to describe cytotoxic T-cell infiltration, regulatory T-cell density, macrophage density, immune checkpoint marker expression, and TAMG-related B-cell/germinal-center features in thymic epithelial tumors (16).

PD-L1 expression was assessed using the tumor proportion score, defined as the percentage of viable tumor cells displaying partial or complete membranous PD-L1 staining at any intensity among all viable tumor cells (17). Cases with fewer than 100 viable tumor cells on the PD-L1 slide were deemed unevaluable for PD-L1-specific analysis.

### 2.11 Composite immune-enriched phenotype

A composite immune-enriched phenotype was prespecified as a secondary exploratory endpoint. This phenotype was considered present when at least two of the following three features were observed: FOXP3-positive T-cell density above the cohort median, CD163-positive macrophage density above the cohort median, and PD-L1 tumor proportion score above the cohort median. CD8-positive T-cell density was analyzed separately and was excluded from this composite definition because CD8-positive T cells in thymoma may include abundant thymocyte-lineage cells and cytotoxic infiltrates with different biological meanings, making median-based inclusion in an immunosuppressive composite difficult to interpret. This composite endpoint served as a descriptive summary of concurrent enrichment of selected immune and immune-checkpoint features and was not interpreted as definitive evidence of functional immunosuppression.

CD20 and CXCL13 were not included in the composite immune-enriched phenotype. They were analyzed as separate exploratory TAMG-related B-cell/germinal-center markers to avoid combining lymphoid-organizing features with the FOXP3/CD163/PD-L1 immune-checkpoint-oriented phenotype.

### 2.12 Outcomes

The prespecified primary pathological endpoint was tumor necrosis proportion. The primary molecular endpoint comprised HIF-1α H-score, and the primary immune endpoint was CD163-positive macrophage density. Secondary endpoints included CAIX H-score, VEGF-A H-score, CD8-positive T-cell density, FOXP3-positive T-cell density, PD-L1 tumor proportion score, CD8/FOXP3 ratio, and the composite immune-enriched phenotype. Exploratory TAMG-related immune endpoints included CD20-positive B-cell density and CXCL13-positive area when evaluable tissue was available.

Procedural safety outcomes encompassed embolization-related chest pain, fever, access-site hematoma, non-target embolization, respiratory deterioration, and transient worsening of myasthenia gravis occurring within 72 hours after embolization. Perioperative outcomes included intraoperative blood loss, conversion to open surgery, postoperative myasthenic crisis, intensive-care-unit admission, and length of postoperative hospital stay. Exploratory MG-related clinical outcomes included postoperative respiratory deterioration, postoperative intravenous immunoglobulin or plasma exchange, first postoperative QMG score, 3-month QMG score when available, and postoperative acetylcholine receptor antibody level when paired laboratory data were available.

### 2.13 Confounding control and bias reduction

Selection bias was mitigated by restricting the analytical cohort to patients who had undergone preoperative embolization and by refraining from direct efficacy comparisons with non-embolized counterparts. To account for potential confounding by tumor biology, tumor maximum diameter, WHO B2/B3 histology, and TNM stage III/IV disease were prespecified as core adjustment covariates. Confounding related to surgical timing was addressed by constraining the embolization-to-surgery interval to 2–5 days in the primary cohort and including this interval as an adjustment variable in regression models. Potential confounding by myasthenia gravis-directed therapy was addressed through systematic extraction of corticosteroid use, prednisone-equivalent dose, non-corticosteroid immunosuppressant use, intravenous immunoglobulin, plasma exchange, MGFA class, and QMG score. These MG severity and treatment variables were evaluated in sensitivity analyses to avoid overfitting in the primary models.

The inferential scope of this study was confined to associations observed within the embolized thymoma-associated myasthenia gravis population. The analysis was not designed to determine whether embolization improves long-term neurological outcomes, recurrence-free survival, or overall survival.

### 2.14 Blinding, reproducibility, and quality control

Radiologists who interpreted CT enhancement and angiographic tumor blush were blinded to pathological necrosis estimates and immunohistochemical results. Pathologists who evaluated necrosis and immunohistochemical staining were blinded to hemodynamic response data, embolization procedural details, MG severity, MG-directed therapy, and clinical outcomes. For continuous imaging and pathological measurements, interobserver agreement was quantified using the intraclass correlation coefficient; for categorical variables, agreement was assessed with Cohen’s kappa. Any discrepancies were resolved through consensus discussion before final data lock.

To evaluate intraobserver reproducibility, 10% of the cases were randomly selected for repeat digital quantification following a two-week washout interval. All immunohistochemical runs were performed in batches that included both positive and negative control slides. In instances where extensive tissue folding, edge artifact, or insufficient viable tumor area compromised assessment, the corresponding blocks were re-cut and restained whenever additional tissue material remained available. Cases remained evaluable for a given marker only when staining quality, tissue preservation, and viable tumor area were sufficient for that marker-specific assessment.

### 2.15 Sample size consideration

The final sample size was governed by the number of eligible patients accrued during the study period and the anticipated association between angiographic hemodynamic response and tumor necrosis proportion. Assuming a two-sided α of 0.05, statistical power of 80%, and a moderate expected correlation coefficient of 0.35 between tumor blush reduction and necrosis proportion, a minimum of 62 evaluable patients was required. Given that thymoma-associated myasthenia gravis managed with preoperative embolization constitutes an uncommon clinical entity, no formal training-validation data split was performed. Instead, all patients with complete core imaging and pathology data were included in the primary analysis.

### 2.16 Statistical analysis

Continuous variables were summarized as mean ± standard deviation or as median with interquartile range, depending on distributional characteristics. Categorical variables were presented as counts and percentages. Normality was assessed through inspection of histograms, Q-Q plots, and the Shapiro–Wilk test. For between-group comparisons across the low-, intermediate-, and high-response strata, one-way analysis of variance or the Kruskal–Wallis test was applied for continuous variables, and the χ² test or Fisher’s exact test was used for categorical variables, as dictated by data type and distribution. Linear trends across the ordered hemodynamic response categories were evaluated using contrast tests for normally distributed variables and the Jonckheere–Terpstra test for non-normally distributed variables. The statistical test used for each variable was reported in the corresponding table or table footnote.

To examine bivariate associations, Spearman correlation analysis was performed among angiographic tumor blush reduction, pre-embolization tumor enhancement, necrosis proportion, HIF-1α H-score, CAIX H-score, VEGF-A H-score, CD8-positive T-cell density, FOXP3-positive T-cell density, CD163-positive macrophage density, PD-L1 tumor proportion score, CD20-positive B-cell density, and CXCL13-positive area when available. The principal visualization outputs comprised box plots, violin plots, scatter plots overlaid with locally estimated smoothing curves, correlation heatmaps, and coefficient plots.

Multivariable linear regression was employed to evaluate the independent association between angiographic hemodynamic response and continuous outcome measures, including necrosis proportion, HIF-1α H-score, and CD163-positive macrophage density. For the composite immune-enriched phenotype, multivariable logistic regression was applied; given the limited number of patients with this composite phenotype, these logistic models were exploratory. The core adjustment set comprised tumor maximum diameter, WHO B2/B3 histology, TNM stage III/IV disease, and embolization-to-surgery interval. Additional covariates—namely age, sex, baseline QMG score, corticosteroid use, prednisone-equivalent dose, non-corticosteroid immunosuppressant use, intravenous immunoglobulin, and plasma exchange—were examined in sensitivity analyses. Regression assumptions were verified through residual plots, variance inflation factor assessment, and influence diagnostics; robust standard errors were applied when heteroscedasticity was detected.

A prespecified exploratory path-oriented analysis was conducted in sequential steps. The first step assessed the association between angiographic hemodynamic response and necrosis proportion. The second step examined the relationship between necrosis proportion and HIF-1α H-score. The third step explored the association between HIF-1α H-score and immune microenvironment markers, with additional adjustment for necrosis proportion and tumor stage. The attenuation of the association between angiographic hemodynamic response and HIF-1α H-score after adjustment for necrosis proportion was interpreted as compatible with a necrosis-linked peri-necrotic hypoxia pathway, without formal causal mediation claims.

Handling of missing data was prespecified before analysis. Variables with more than 20% missingness were excluded from multivariable modeling. For variables with missingness of 5% or less, complete-case analysis was applied. For variables with missingness exceeding 5% but not surpassing 20%, multiple imputation by chained equations was performed, generating 20 imputed datasets under the clinically plausible assumption of missing at random. Complete-case results were additionally reported as sensitivity analyses.

Because multiple tissue-level endpoints were evaluated, Benjamini–Hochberg false-discovery-rate adjustment was performed as a sensitivity analysis for the main pathological, hypoxia-related, and immune-marker comparisons. Unadjusted P values, FDR-adjusted q values, effect sizes, and 95% confidence intervals were reported together. All statistical computations were carried out using R version 4.5.3. A two-sided P value <0.05 was considered statistically significant, with emphasis placed on effect-size direction, confidence intervals, and consistency across sensitivity analyses.

## 3 Results

### 3.1 Patient selection and analytical cohort

Between January 2018 and June 2025, 214 patients undergoing surgical resection for thymic epithelial tumors were screened for eligibility. Of these, 52 were excluded because the final pathological diagnosis was not thymoma: thymic carcinoma (n = 30), thymic neuroendocrine tumor (n = 7), lymphoma or germ-cell tumor (n = 6), metastatic tumor (n = 3), and non-neoplastic thymic disease (n = 6). This left 162 patients with histologically confirmed thymoma. Among them, 81 patients had sporadic thymoma without clinical myasthenia gravis and were not included because the present analysis focused on embolized thymoma-associated myasthenia gravis. The remaining 81 patients had thymoma-associated myasthenia gravis.

Before embolization, 13 patients were excluded: 9 because the tumor lacked hypervascularity on CT angiography and 4 because safe superselective embolization was not technically feasible. Thus, 68 patients underwent clinically indicated preoperative transarterial embolization. After embolization, 4 patients were excluded: surgery was delayed beyond 7 days in 2 cases, paired digital subtraction angiography images were incomplete in 1 case, and residual viable tumor tissue was insufficient for immunohistochemical analysis in 1 case. The final analytical cohort comprised 64 patients with complete core angiographic, pathological, and immunohistochemical data.

Based on angiographic tumor blush reduction, 19 patients were classified into the low-response group, 27 into the intermediate-response group, and 18 into the high-response group. The mean interval from embolization to surgical resection was 3.2 ± 0.9 days.

### 3.2 Baseline clinical, neurological, and tumor characteristics

Baseline demographic, neurological, treatment, and tumor-related variables are presented in **Table 1**. The overall mean age was 52.8 ± 12.7 years, and 27 patients were female. With respect to myasthenia gravis, 46 patients had MGFA class II–III disease, the mean QMG score was 14.6 ± 4.1, and serum acetylcholine receptor antibodies were positive in 58 patients. MG-directed therapy before surgery included pyridostigmine in 56 patients, corticosteroids in 31 patients, non-corticosteroid immunosuppressants in 14 patients, intravenous immunoglobulin in 12 patients, and plasma exchange in 5 patients.

**Table 1:** Baseline characteristics according to angiographic hemodynamic response.

Variable	Overall *n* = 64	Low response *n* = 19	Intermediate response *n* = 27	High response *n* = 18	P value
Age, years	52.8 ± 12.7	54.7 ± 10.9	50.6 ± 14.0	53.9 ± 12.8	0.535
Female sex	27/64 (42.2%)	7/19 (36.8%)	12/27 (44.4%)	8/18 (44.4%)	0.846
Body mass index, kg/m²	23.9 ± 3.0	24.4 ± 3.3	23.5 ± 2.7	24.1 ± 3.2	0.604
Hypertension	18/64 (28.1%)	6/19 (31.6%)	7/27 (25.9%)	5/18 (27.8%)	0.912
Diabetes mellitus	9/64 (14.1%)	4/19 (21.1%)	3/27 (11.1%)	2/18 (11.1%)	0.574
Coronary artery disease	4/64 (6.3%)	1/19 (5.3%)	2/27 (7.4%)	1/18 (5.6%)	0.948
MG duration, months	8.5 [3.8–18.0]	8.1 [3.2–17.4]	9.3 [4.5–20.5]	7.6 [3.6–16.8]	0.741
MGFA class II–III	46/64 (71.9%)	14/19 (73.7%)	18/27 (66.7%)	14/18 (77.8%)	0.714
QMG score	14.6 ± 4.1	14.9 ± 4.4	14.1 ± 3.9	15.0 ± 4.2	0.735
AChR antibody positive	58/64 (90.6%)	17/19 (89.5%)	24/27 (88.9%)	17/18 (94.4%)	0.820
AChR antibody, nmol/L	9.4 [4.8–17.6]	9.1 [4.3–19.2]	8.8 [4.9–16.5]	10.6 [5.3–18.7]	0.692
MuSK antibody tested	41/64 (64.1%)	12/19 (63.2%)	17/27 (63.0%)	12/18 (66.7%)	0.963
MuSK antibody positive	2/41 (4.9%)	0/12 (0.0%)	1/17 (5.9%)	1/12 (8.3%)	0.591
Pyridostigmine use	56/64 (87.5%)	16/19 (84.2%)	24/27 (88.9%)	16/18 (88.9%)	0.867
Corticosteroid use	31/64 (48.4%)	10/19 (52.6%)	12/27 (44.4%)	9/18 (50.0%)	0.856
Prednisone-equivalent dose, mg/day	10 [5–20]	10 [5–20]	10 [5–15]	12.5 [5–20]	0.706
Non-corticosteroid immunosuppressant use	14/64 (21.9%)	5/19 (26.3%)	5/27 (18.5%)	4/18 (22.2%)	0.813
Intravenous immunoglobulin	12/64 (18.8%)	4/19 (21.1%)	4/27 (14.8%)	4/18 (22.2%)	0.763
Plasma exchange	5/64 (7.8%)	2/19 (10.5%)	1/27 (3.7%)	2/18 (11.1%)	0.554
Tumor maximum diameter, cm	6.6 ± 1.6	6.4 ± 1.8	6.8 ± 1.5	6.5 ± 1.7	0.684
WHO B2/B3 histology	39/64 (60.9%)	12/19 (63.2%)	15/27 (55.6%)	12/18 (66.7%)	0.737
TNM stage III/IV disease	20/64 (31.3%)	7/19 (36.8%)	7/27 (25.9%)	6/18 (33.3%)	0.711
Pre-embolization tumor enhancement, HU	118.6 ± 24.2	121.4 ± 26.1	115.7 ± 23.8	120.3 ± 22.9	0.711

AChR, acetylcholine receptor; HU, Hounsfield unit; MG, myasthenia gravis; MGFA, Myasthenia Gravis Foundation of America; MuSK, muscle-specific kinase; QMG, Quantitative Myasthenia Gravis; WHO, World Health Organization. Normally distributed continuous variables were compared using one-way ANOVA, skewed continuous variables using the Kruskal–Wallis test, and categorical variables using the χ² test or Fisher’s exact test.

The mean maximum tumor diameter was 6.6 ± 1.6 cm. WHO B2/B3 histology was identified in 39 patients, and TNM stage III/IV disease was documented in 20 patients. Across the three hemodynamic response strata, baseline MG severity, AChR antibody level, MG-directed therapy, tumor size, WHO B2/B3 histology, TNM stage III/IV disease, and pre-embolization tumor enhancement showed no clear between-group imbalance.

### 3.3 Embolization procedure, hemodynamic response, and perioperative features

Procedural and perioperative characteristics are detailed in **Table 2**. The internal thoracic artery or its branches were the most common feeding vessels. Additional supply from pericardiophrenic or other small mediastinal branches was observed in 29 patients, and 36 patients had multiple feeding arteries. Polyvinyl alcohol particles were used in all cases; supplemental gelatin sponge particles were added in 21 patients, and coils were deployed in 7 patients.

**Table 2:** Embolization procedure, perioperative features, and exploratory early MG outcomes.

Variable	Overall *n* = 64	Low response *n* = 19	Intermediate response *n* = 27	High response *n* = 18	P value
Internal thoracic artery feeder	42/64 (65.6%)	13/19 (68.4%)	17/27 (63.0%)	12/18 (66.7%)	0.929
Pericardiophrenic/mediastinal feeder	29/64 (45.3%)	9/19 (47.4%)	11/27 (40.7%)	9/18 (50.0%)	0.817
Bronchial artery feeder	17/64 (26.6%)	6/19 (31.6%)	6/27 (22.2%)	5/18 (27.8%)	0.775
Intercostal artery feeder	10/64 (15.6%)	4/19 (21.1%)	3/27 (11.1%)	3/18 (16.7%)	0.651
Multiple feeding arteries	36/64 (56.3%)	10/19 (52.6%)	16/27 (59.3%)	10/18 (55.6%)	0.898
Polyvinyl alcohol particles	64/64 (100.0%)	19/19 (100.0%)	27/27 (100.0%)	18/18 (100.0%)	—
Additional gelatin sponge particles	21/64 (32.8%)	5/19 (26.3%)	8/27 (29.6%)	8/18 (44.4%)	0.428
Coil use	7/64 (10.9%)	2/19 (10.5%)	4/27 (14.8%)	1/18 (5.6%)	0.618
Technical success	61/64 (95.3%)	17/19 (89.5%)	26/27 (96.3%)	18/18 (100.0%)	0.228
Angiographic tumor blush reduction, %	56.2 [42.6–68.4]	35.6 [29.8–40.9]	55.0 [50.2–60.7]	72.8 [68.2–78.9]	<0.001
Embolization-to-surgery interval, days	3.2 ± 0.9	3.1 ± 0.8	3.4 ± 0.9	3.0 ± 0.9	0.267
VATS or robotic surgery	33/64 (51.6%)	8/19 (42.1%)	16/27 (59.3%)	9/18 (50.0%)	0.514
Sternotomy/thoracotomy/combined approach	31/64 (48.4%)	11/19 (57.9%)	11/27 (40.7%)	9/18 (50.0%)	0.514
R0 resection	58/64 (90.6%)	16/19 (84.2%)	25/27 (92.6%)	17/18 (94.4%)	0.501
Intraoperative blood loss, mL	250 [170–380]	310 [210–460]	240 [170–370]	220 [150–340]	0.046
Postoperative myasthenic crisis	6/64 (9.4%)	2/19 (10.5%)	3/27 (11.1%)	1/18 (5.6%)	0.814
Postoperative respiratory deterioration	9/64 (14.1%)	4/19 (21.1%)	3/27 (11.1%)	2/18 (11.1%)	0.574
Postoperative intravenous immunoglobulin or plasma exchange	8/64 (12.5%)	3/19 (15.8%)	4/27 (14.8%)	1/18 (5.6%)	0.569
ICU admission	16/64 (25.0%)	6/19 (31.6%)	7/27 (25.9%)	3/18 (16.7%)	0.564
Postoperative hospital stay, days	9 [7–12]	10 [8–13]	9 [7–12]	8 [7–11]	0.173

ICU, intensive-care unit; MG, myasthenia gravis; VATS, video-assisted thoracoscopic surgery. Normally distributed continuous variables were compared using one-way ANOVA, skewed continuous variables using the Kruskal–Wallis test, and categorical variables using the χ² test or Fisher’s exact test.

**Figure 2:**
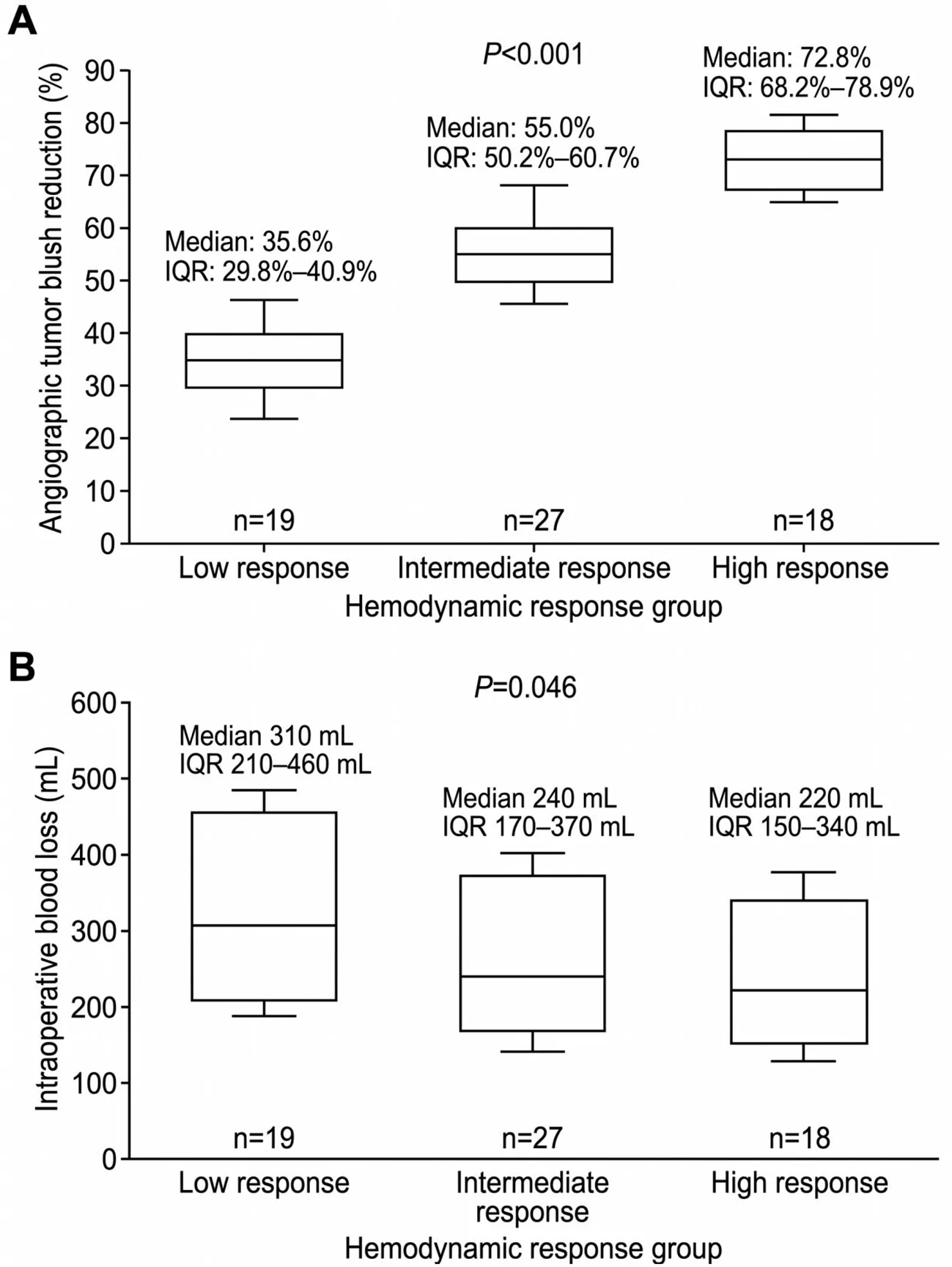
Angiographic hemodynamic response and intraoperative blood loss. **(A)** Distribution of angiographic tumor blush reduction across the low-, intermediate-, and high-response groups. **(B)** Intraoperative blood loss across the three response groups. Boxes indicate interquartile ranges, central lines indicate medians, and individual points indicate patients.

The overall median angiographic tumor blush reduction was 56.2%. By design, blush reduction differed across the low-, intermediate-, and high-response groups. Embolization-to-surgery interval, operative approach, and R0 resection rate were similar across response strata. Intraoperative blood loss was lower in the high-response group in the unadjusted comparison, although the distributions overlapped across groups. Early postoperative MG-related outcomes, including postoperative myasthenic crisis, respiratory deterioration, and postoperative intravenous immunoglobulin or plasma exchange, showed no clear separation across hemodynamic response strata. The relationship between angiographic tumor blush reduction and intraoperative blood loss is displayed in **Figure 2**.

### 3.4 Tumor necrosis, hypoxia-related markers, and immune microenvironment findings

**Figure 3:**
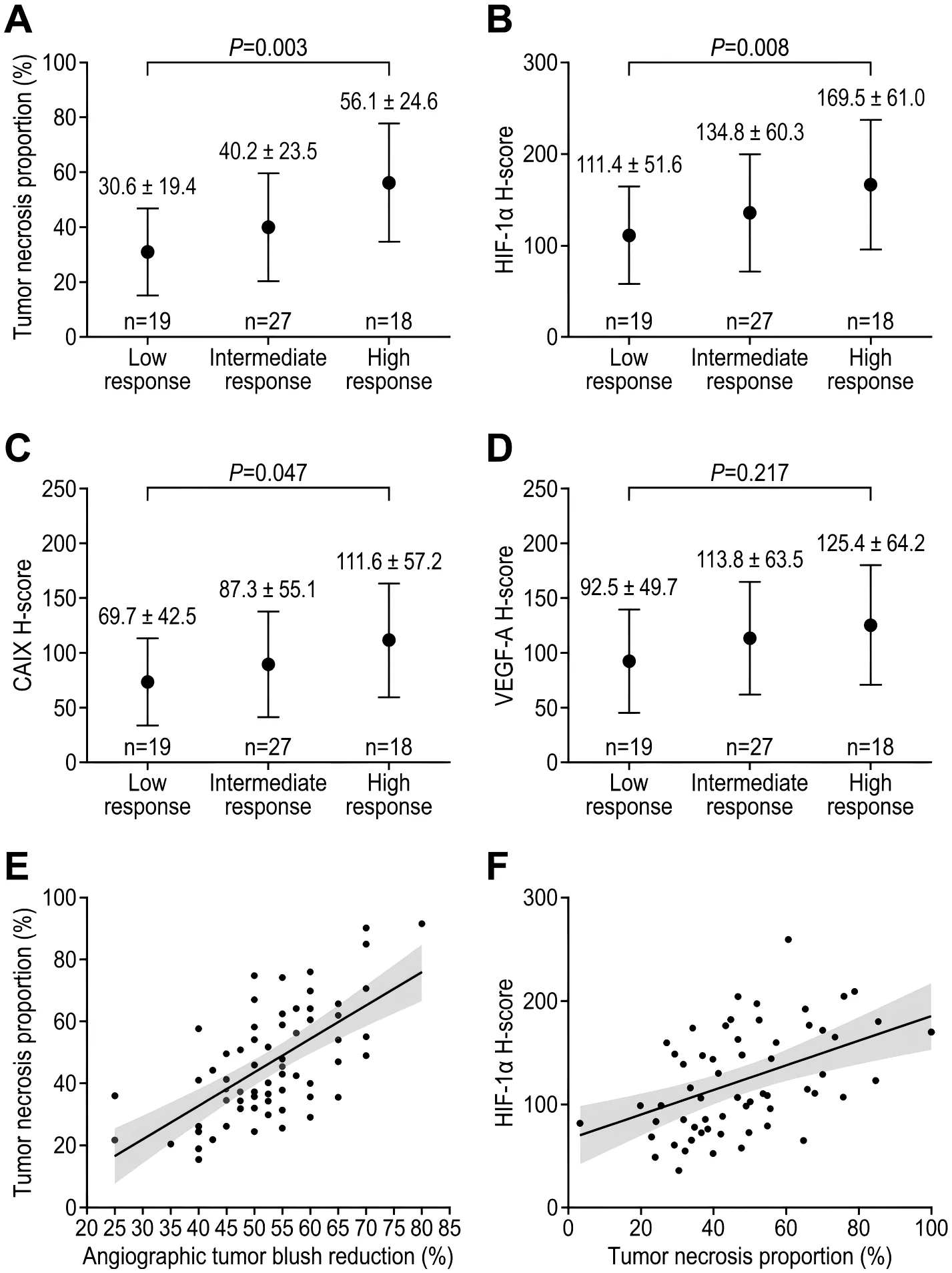
Tumor necrosis and hypoxia-related marker expression. **(A)** Tumor necrosis proportion across hemodynamic response groups. **(B)** HIF-1α H-score across response groups. **(C)** CAIX H-score across response groups. **(D)** VEGF-A H-score across response groups. **(E)** Association between angiographic tumor blush reduction and tumor necrosis proportion. **(F)** Association between tumor necrosis proportion and HIF-1α H-score. Angiographic tumor blush reduction was positively correlated with tumor necrosis proportion and HIF-1α H-score. Correlations with CAIX and VEGF-A were weaker. Necrosis proportion was moderately correlated with HIF-1α H-score.

The distributions of tumor necrosis proportion, hypoxia-related marker expression, and immune microenvironment variables are summarized in **Table 3**. Tumor necrosis proportion increased across response strata, with the highest value in the high-response group. HIF-1α H-score showed a similar graded pattern. These between-group distributions, together with the correlations between angiographic tumor blush reduction, tumor necrosis proportion, and HIF-1α H-score, are illustrated in **Figure 3**. These two endpoints remained below the false-discovery-rate threshold in the sensitivity analysis. CAIX H-score and CD163-positive macrophage density showed unadjusted between-group differences, but their FDR-adjusted q values did not remain below 0.05. VEGF-A, CD8-positive T-cell density, FOXP3-positive T-cell density, PD-L1 tumor proportion score, CD8/FOXP3 ratio, and the composite immune-enriched phenotype showed broad overlap across groups.

**Table 3:** Pathological, hypoxia-related, and immune microenvironment findings.

Variable	Overall n=64	Low response n=19	Intermediate response n=27	High response n=18	Test used	P value	FDR q value
Tumor necrosis proportion, %	42.1 ± 23.8	30.6 ± 19.4	40.2 ± 23.5	56.1 ± 24.6	ANOVA	0.003	0.036
HIF-1α H-score	137.6 ± 61.2	111.4 ± 51.6	134.8 ± 60.3	169.5 ± 61.0	ANOVA	0.008	0.048
CAIX H-score	88.9 ± 53.4	69.7 ± 42.5	87.3 ± 55.1	111.6 ± 57.2	ANOVA	0.047	0.141
VEGF-A H-score	110.4 ± 60.8	92.5 ± 49.7	113.8 ± 63.5	125.4 ± 64.2	ANOVA	0.217	0.434
CD8-positive T cells, cells/mm²	96.8 ± 50.6	89.2 ± 42.7	103.5 ± 55.1	94.8 ± 51.6	ANOVA	0.639	0.767
FOXP3-positive T cells, cells/mm²	31.7 ± 21.4	26.8 ± 17.3	30.5 ± 22.6	38.2 ± 23.1	ANOVA	0.284	0.487
CD163-positive macrophages, cells/mm²	62.5 ± 34.9	49.5 ± 27.8	60.4 ± 35.7	79.2 ± 37.1	ANOVA	0.036	0.141
PD-L1 TPS, %	31.6 ± 26.2	26.4 ± 22.6	30.8 ± 25.7	38.1 ± 30.1	ANOVA	0.392	0.588
CD8/FOXP3 ratio	3.6 ± 3.0	3.8 ± 3.2	3.9 ± 3.1	3.0 ± 2.5	ANOVA	0.589	0.767
Composite immune-enriched phenotype	24/64 (37.5%)	5/19 (26.3%)	9/27 (33.3%)	10/18 (55.6%)	Fisher’s exact	0.167	0.401
CD20-positive B cells, cells/mm²	44.6 ± 29.8	48.2 ± 31.1	42.7 ± 28.6	43.9 ± 30.4	ANOVA	0.842	0.901
CXCL13-positive area, %	6.8 [3.1–11.4]	7.2 [3.2–10.9]	6.5 [2.8–12.1]	7.0 [3.4–11.0]	Kruskal–Wallis	0.901	0.901

CAIX, carbonic anhydrase IX; FDR, false discovery rate; HIF-1α, hypoxia-inducible factor-1α; PD-L1, programmed death-ligand 1; TPS, tumor proportion score; VEGF-A, vascular endothelial growth factor A. FDR q values were calculated using the Benjamini–Hochberg method across the tissue-level endpoints listed in this table. CD20 and CXCL13 analyses were exploratory and based on evaluable residual tissue: overall n=58, low response n=17, intermediate response n=25, and high response n=16.

Exploratory CD20 and CXCL13 analyses were evaluable in residual tissue from 58 patients. CD20-positive B-cell density and CXCL13-positive area did not show a graded increase across hemodynamic response strata. Collectively, these findings indicate that stronger angiographic devascularization was most consistently associated with ischemic necrosis and HIF-1α expression, whereas immune-marker patterns were more heterogeneous.

### 3.5 Immune microenvironment features

**Figure 4:**
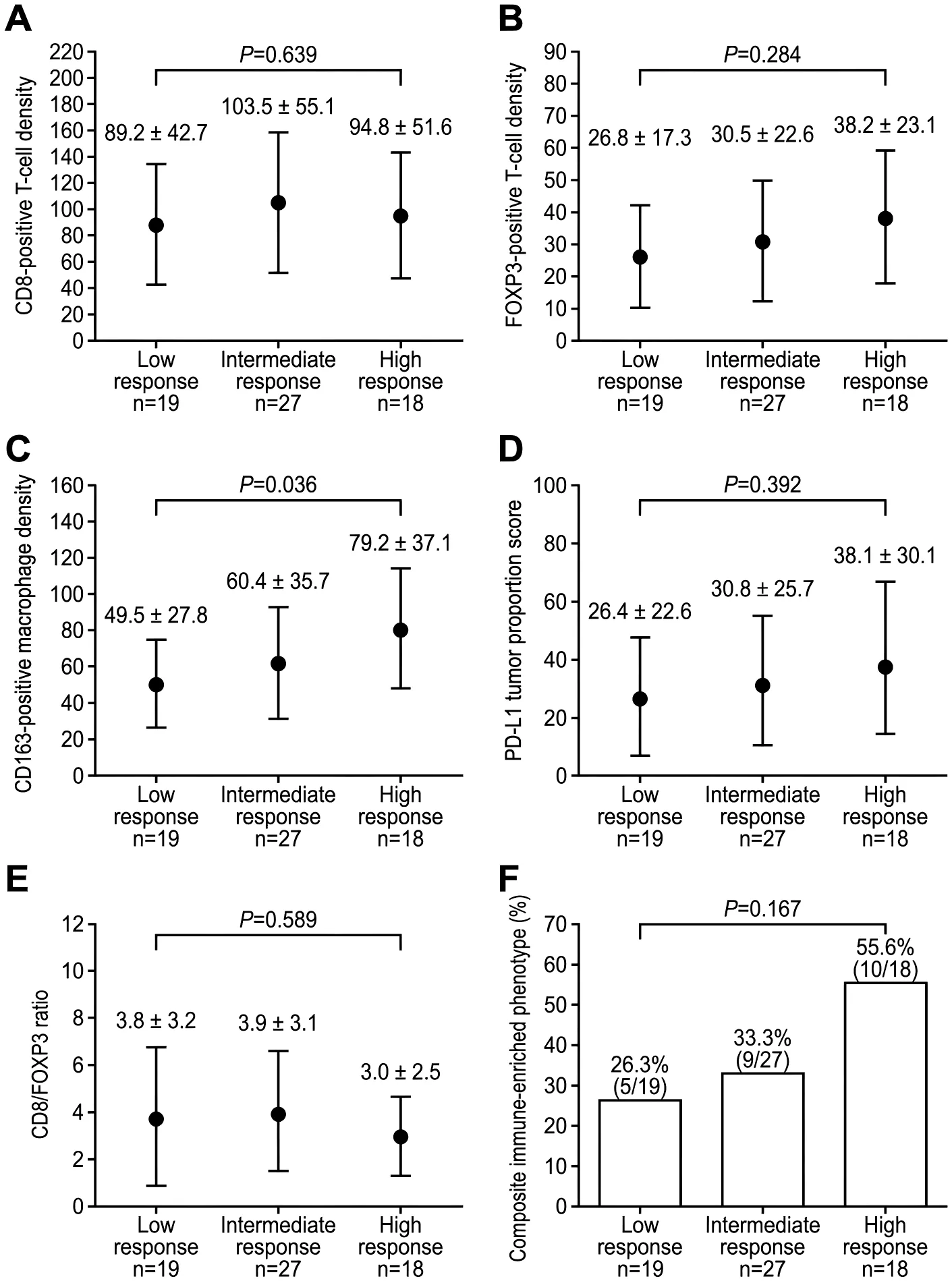
Immune microenvironment features across hemodynamic response groups. **(A)** CD8-positive T-cell density. **(B)** FOXP3-positive T-cell density. **(C)** CD163-positive macrophage density. **(D)** PD-L1 tumor proportion score. **(E)** CD8/FOXP3 ratio. **(F)** Frequency of the composite immune-enriched phenotype. Exploratory CD20-positive B-cell density and CXCL13-positive area are summarized in **Table 3**.

Immune microenvironment findings are shown in **Table 3** and **Figure 4**. CD163-positive macrophage density was highest in the high-response group in the unadjusted comparison. However, after false-discovery-rate adjustment across tissue-level endpoints, this difference was interpreted as an exploratory immune signal rather than a definitive group-level finding. FOXP3-positive regulatory T-cell density was numerically higher in the high-response group, but the difference was not statistically significant. CD8-positive T-cell density, PD-L1 tumor proportion score, and the CD8/FOXP3 ratio showed broad overlap across response strata.

Exploratory B-cell and germinal-center-related markers were evaluable in residual tissue from 58 patients. CD20-positive B-cell density and CXCL13-positive area did not show a graded increase across the low-, intermediate-, and high-response groups. These findings suggest that the strongest tissue-level changes after greater angiographic devascularization were related to necrosis and HIF-1α expression, whereas TAMG-related lymphoid-organizing features were not clearly separated by hemodynamic response.

The composite immune-enriched phenotype was identified in 24 patients overall. Its frequency was 26.3% in the low-response group, 33.3% in the intermediate-response group, and 55.6% in the high-response group, but this gradient did not reach statistical significance. The median threshold values used to define the composite phenotype were 29.8 cells/mm² for FOXP3-positive T-cell density, 59.6 cells/mm² for CD163-positive macrophage density, and 28.0% for PD-L1 tumor proportion score. CD8-positive T-cell density was not included in the composite phenotype because it was analyzed as a separate cytotoxic/lymphocyte-lineage marker. CD20 and CXCL13 were also analyzed separately as exploratory TAMG-related B-cell and germinal-center markers.

### 3.6 Multivariable association models

**Figure 5:**
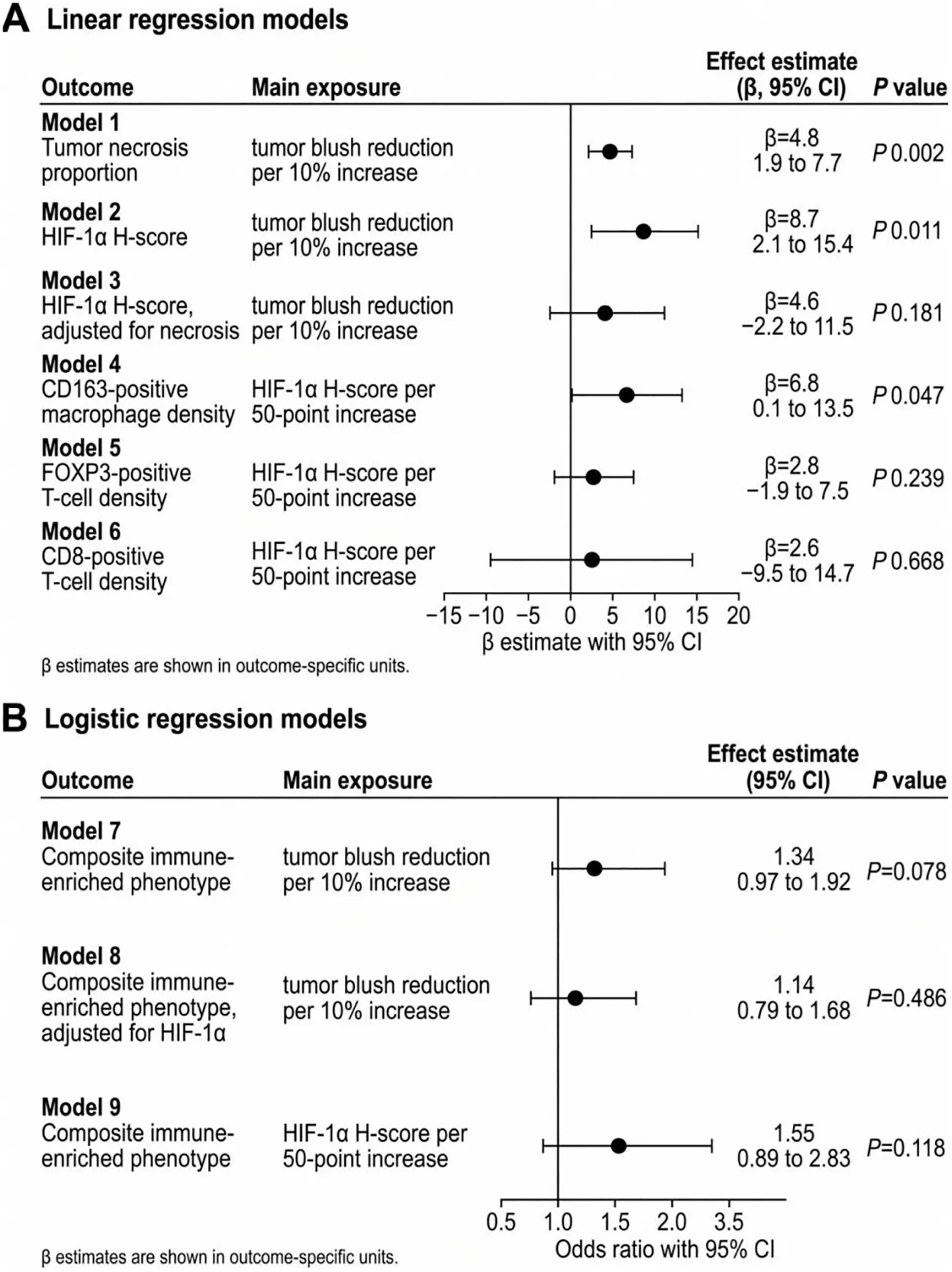
Multivariable association models. **(A)** Linear regression estimates for tumor necrosis proportion, HIF-1α H-score, and immune-cell density outcomes. **(B)** Logistic regression estimates for the composite immune-enriched phenotype. Effect estimates are shown with 95% confidence intervals.

The results of the multivariable regression models are summarized in **Table 4**, with corresponding effect estimates and 95% confidence intervals shown in **Figure 5**.

**Table 4:** Multivariable association models.

Model	Outcome	Main exposure	Adjustment variables	Effect estimate	P value
Model 1	Tumor necrosis proportion	Tumor blush reduction, per 10% increase	Tumor maximum diameter, WHO B2/B3 histology, TNM stage III/IV disease, embolization-to-surgery interval	β = 4.8 percentage points, 95% CI 1.9–7.7	0.002
Model 2	HIF-1α H-score	Tumor blush reduction, per 10% increase	Tumor maximum diameter, WHO B2/B3 histology, TNM stage III/IV disease, embolization-to-surgery interval	β = 8.7, 95% CI 2.1–15.4	0.011
Model 3	HIF-1α H-score	Tumor blush reduction, per 10% increase	Model 2 variables plus tumor necrosis proportion	β = 4.6, 95% CI −2.2 to 11.5	0.181
Model 4	CD163-positive macrophage density	HIF-1α H-score, per 50-point increase	Tumor maximum diameter, TNM stage III/IV disease, tumor necrosis proportion	β = 6.8 cells/mm², 95% CI 0.1–13.5	0.047
Model 5	FOXP3-positive T-cell density	HIF-1α H-score, per 50-point increase	Tumor maximum diameter, TNM stage III/IV disease, tumor necrosis proportion	β = 2.8 cells/mm², 95% CI −1.9 to 7.5	0.239
Model 6	CD8-positive T-cell density	HIF-1α H-score, per 50-point increase	Tumor maximum diameter, TNM stage III/IV disease, tumor necrosis proportion	β = 2.6 cells/mm², 95% CI −9.5 to 14.7	0.668
Model 7	Composite immune-enriched phenotype	Tumor blush reduction, per 10% increase	Tumor maximum diameter, WHO B2/B3 histology, TNM stage III/IV disease, embolization-to-surgery interval	OR = 1.34, 95% CI 0.97–1.92	0.078
Model 8	Composite immune-enriched phenotype	Tumor blush reduction, per 10% increase	Model 7 variables plus HIF-1α H-score	OR = 1.14, 95% CI 0.79–1.68	0.486
Model 9	Composite immune-enriched phenotype	HIF-1α H-score, per 50-point increase	Tumor maximum diameter, WHO B2/B3 histology, TNM stage III/IV disease, embolization-to-surgery interval	OR = 1.55, 95% CI 0.89–2.83	0.118
Model 10	CD20-positive B-cell density	Tumor blush reduction, per 10% increase	Tumor maximum diameter, WHO B2/B3 histology, TNM stage III/IV disease, embolization-to-surgery interval	β = −0.7 cells/mm², 95% CI −4.1 to 2.8	0.690
Model 11	CXCL13-positive area	Tumor blush reduction, per 10% increase	Tumor maximum diameter, WHO B2/B3 histology, TNM stage III/IV disease, embolization-to-surgery interval	β = 0.2 percentage points, 95% CI −0.6 to 1.1	0.598

CI, confidence interval; HIF-1α, hypoxia-inducible factor-1α; OR, odds ratio; WHO, World Health Organization. Models 10 and 11 were exploratory and were based on cases with evaluable residual tissue for CD20 and CXCL13 staining.

After adjustment for tumor maximum diameter, WHO B2/B3 histology, TNM stage III/IV disease, and embolization-to-surgery interval, each 10% increase in angiographic tumor blush reduction was associated with a 4.8 percentage-point increase in tumor necrosis proportion. Tumor blush reduction was also associated with higher HIF-1α H-score in the core adjusted model. After additional adjustment for tumor necrosis proportion, the association between tumor blush reduction and HIF-1α H-score was attenuated and no longer statistically significant. This pattern was compatible with necrosis-linked peri-necrotic hypoxia contributing to HIF-1α expression, although formal mediation was not inferred.

For immune markers, HIF-1α H-score showed a weak positive association with CD163-positive macrophage density after adjustment for tumor maximum diameter, TNM stage III/IV disease, and tumor necrosis proportion. Associations with FOXP3-positive T-cell density and CD8-positive T-cell density were not statistically significant. In exploratory logistic regression, tumor blush reduction showed a non-significant association with the composite immune-enriched phenotype in the core adjusted model, and this association was attenuated after inclusion of HIF-1α H-score. Exploratory models for CD20-positive B-cell density and CXCL13-positive area did not show significant associations with tumor blush reduction.

### 3.7 Sensitivity analyses and reproducibility

In sensitivity analyses incorporating additional adjustment for age, sex, baseline QMG score, corticosteroid use, prednisone-equivalent dose, non-corticosteroid immunosuppressant use, intravenous immunoglobulin, and plasma exchange, the association between tumor blush reduction and tumor necrosis proportion remained statistically significant. The association between tumor blush reduction and HIF-1α H-score remained positive in direction but was reduced after adjustment for necrosis proportion. The association between HIF-1α H-score and CD163-positive macrophage density remained similar in direction, although the confidence interval widened. Complete-case analyses yielded effect estimates comparable in direction to those from the primary imputed models.

Among variables included in the primary multivariable models, none had missingness exceeding 20%. MuSK antibody status was not tested in all patients and was used only for descriptive analysis. Postoperative QMG score and postoperative acetylcholine receptor antibody level were available only in subsets of patients and were therefore analyzed as exploratory available-case outcomes. Variance inflation factors for the primary models were all below 2.5.

Interobserver reproducibility was acceptable across key measurements. The intraclass correlation coefficients were 0.86 for angiographic tumor blush reduction, 0.82 for tumor necrosis proportion, 0.84 for HIF-1α H-score, and 0.80 for CD163-positive macrophage density. For exploratory CD20-positive B-cell density and CXCL13-positive area, intraclass correlation coefficients were 0.81 and 0.78, respectively. For the composite immune-enriched phenotype, Cohen’s kappa was 0.76. Intraobserver reproducibility, assessed by repeat digital quantification in 10% of randomly selected cases, yielded intraclass correlation coefficients ranging from 0.79 to 0.89.

### 3.8 Procedural safety and postoperative MG outcomes

Safety and exploratory postoperative MG outcomes are summarized in **Tables 5**, **6**. Embolization-related adverse events were mostly mild. At least one embolization-related adverse event occurred in 20 patients, with transient chest discomfort or low-grade fever being the most common manifestation. Access-site hematoma developed in two patients, both managed conservatively. Transient worsening of MG symptoms within 72 hours after embolization was observed in three patients. No non-target embolization, spinal ischemia, stroke, myocardial infarction, procedure-related death, or in-hospital death was recorded.

**Table 5:** Embolization-related and postoperative safety outcomes.

Variable	Overall *n* = 64	Low response *n* = 19	Intermediate response *n* = 27	High response *n* = 18	P value
Any embolization-related adverse event	20/64 (31.3%)	6/19 (31.6%)	7/27 (25.9%)	7/18 (38.9%)	0.653
Chest pain or low-grade fever	18/64 (28.1%)	5/19 (26.3%)	7/27 (25.9%)	6/18 (33.3%)	0.842
Access-site hematoma	2/64 (3.1%)	1/19 (5.3%)	1/27 (3.7%)	0/18 (0.0%)	0.606
Transient MG worsening within 72 hours	3/64 (4.7%)	1/19 (5.3%)	1/27 (3.7%)	1/18 (5.6%)	0.965
Non-target embolization	0/64 (0.0%)	0/19 (0.0%)	0/27 (0.0%)	0/18 (0.0%)	—
Postoperative myasthenic crisis	5/64 (7.8%)	2/19 (10.5%)	1/27 (3.7%)	2/18 (11.1%)	0.554
ICU admission	15/64 (23.4%)	6/19 (31.6%)	5/27 (18.5%)	4/18 (22.2%)	0.568
In-hospital death	0/64 (0.0%)	0/19 (0.0%)	0/27 (0.0%)	0/18 (0.0%)	—

ICU, intensive-care unit; MG, myasthenia gravis. Categorical variables were compared using the χ² test or Fisher’s exact test.

**Table 6:** Exploratory postoperative MG outcomes.

Variable	Overall	Low response	Intermediate response	High response	P value
First postoperative QMG score available	56/64 (87.5%)	17/19 (89.5%)	23/27 (85.2%)	16/18 (88.9%)	0.904
Baseline QMG score in evaluable patients	14.5 ± 4.0	14.8 ± 4.2	14.0 ± 3.8	15.0 ± 4.1	0.748
First postoperative QMG score	11.9 ± 4.3	12.7 ± 4.4	11.5 ± 4.1	11.7 ± 4.5	0.650
Early QMG improvement	2.6 ± 2.5	2.1 ± 2.3	2.5 ± 2.4	3.3 ± 2.7	0.312
Three-month QMG score available	43/64 (67.2%)	12/19 (63.2%)	18/27 (66.7%)	13/18 (72.2%)	0.846
Three-month QMG score	9.6 ± 4.1	10.1 ± 4.4	9.4 ± 3.9	9.3 ± 4.2	0.861
Three-month QMG improvement	4.9 ± 3.1	4.5 ± 3.0	4.8 ± 3.2	5.4 ± 3.2	0.711
Postoperative AChR antibody level available	37/64 (57.8%)	11/19 (57.9%)	16/27 (59.3%)	10/18 (55.6%)	0.970
Postoperative AChR antibody level, nmol/L	7.8 [3.9–14.6]	7.9 [4.0–15.1]	7.5 [3.6–13.8]	8.2 [4.2–14.9]	0.884
Change in AChR antibody level, nmol/L	−1.1 [−3.8 to 0.6]	−0.9 [−3.5 to 0.8]	−1.2 [−4.0 to 0.5]	−1.0 [−3.7 to 0.7]	0.936

AChR, acetylcholine receptor; MG, myasthenia gravis; QMG, Quantitative Myasthenia Gravis. Early QMG improvement was calculated as baseline QMG score minus first postoperative QMG score. Three-month QMG improvement was calculated as baseline QMG score minus the QMG score closest to 3 months after surgery. Continuous variables were compared using one-way ANOVA or the Kruskal–Wallis test, and categorical variables using the χ² test or Fisher’s exact test.

Postoperative myasthenic crisis occurred in five patients. Intensive-care-unit admission was required in 15 patients, including 9 planned admissions for postoperative monitoring. Across the three hemodynamic response groups, safety outcomes showed no statistically significant differences. Available postoperative neurological data did not show a clear association between greater tumor blush reduction and early MG improvement. QMG score decreased from baseline to the first postoperative reassessment and to approximately 3 months after surgery in the available-case analysis, but the magnitude of change did not differ significantly across response strata. Paired postoperative acetylcholine receptor antibody data were limited and showed no clear graded change by hemodynamic response.

## 4 Discussion

This single-center translational cohort study evaluated patients with thymoma-associated myasthenia gravis who underwent clinically indicated preoperative transarterial embolization, integrating paired angiographic, pathological, and immunohistochemical data. Three findings were most consistent. First, greater angiographic tumor blush reduction was associated with a higher tumor necrosis proportion. Second, tumor blush reduction was associated with higher HIF-1α expression in the core adjusted model, but this association was attenuated after adjustment for necrosis proportion. Third, immune microenvironmental findings were more selective: CD163-positive macrophage density showed the clearest exploratory signal, whereas CD8-positive T-cell density, FOXP3-positive regulatory T-cell density, PD-L1 tumor proportion score, the CD8/FOXP3 ratio, and the composite immune-enriched phenotype showed broader overlap across response groups. Exploratory CD20 and CXCL13 analyses did not show a graded increase across hemodynamic response strata. Overall, angiographic response was more consistently linked to ischemic necrosis and HIF-1α expression than to a uniform shift in the TAMG immune microenvironment.

The association between angiographic devascularization and tumor necrosis is biologically plausible. Preoperative embolization has been used in selected hypervascular thoracic and mediastinal tumors to reduce vascularity before resection; however, the tissue effect may vary according to feeding-artery anatomy, collateral supply, catheter position, embolic distribution, and the interval before surgery (18). In this study, the exposure was not embolization as a binary intervention, because all patients in the analytical cohort underwent embolization. The exposure was the measured degree of tumor blush reduction on completion angiography. This distinction matters. Technical success records that embolization was completed, whereas quantitative blush reduction captures the extent of immediate tumor devascularization. The graded relationship between blush reduction and necrosis proportion therefore supports the use of angiographic response as a procedural readout in future studies of hypervascular thymoma.

The HIF-1α result should be read together with the necrosis finding. HIF-1α is a central marker of cellular adaptation to hypoxia and may be expressed in viable tumor cells near ischemic or necrotic tissue interfaces (19). In the present analysis, the association between blush reduction and HIF-1α weakened after adding necrosis proportion to the model. This pattern suggests that necrosis, or the peri-necrotic hypoxic zone around necrotic tumor bed, may partly account for the HIF-1α signal. It does not prove formal mediation. The absence of a parallel significant VEGF-A increase also has several possible explanations. VEGF-A induction after arterial embolization can be time-dependent, and the 2–5 day sampling window used here may not have captured peak angiogenic signaling in all patients. In addition, thymoma-specific regulation of hypoxia signaling, variable viable tumor fraction, and immunohistochemical sampling of post-embolization tissue may have reduced the ability to detect a VEGF-A difference. Findings from embolization studies in other solid tumors, including hepatocellular carcinoma, also support that HIF-1α and VEGF-related responses can vary by post-procedural timing and residual viable tissue context (20).

The immune findings were less uniform than the ischemic and hypoxia-related findings. CD163-positive macrophage density was highest in the high-response group in the unadjusted comparison, but after false-discovery-rate adjustment this finding was best interpreted as exploratory. CD163 is often used as a marker of M2-like or tissue-remodeling macrophages, but in TAMG its meaning is unlikely to be limited to simple tumor immunosuppression. CD163-positive macrophages in this cohort may reflect ischemia-related recruitment and clearance of necrotic tissue, remodeling around peri-necrotic viable tumor, or pre-existing macrophage niches within the MG-associated thymic microenvironment. These possibilities cannot be separated without pre-embolization tissue or a non-embolized TAMG comparator. Recent spatial and single-cell studies of MG-associated thymoma have emphasized that epithelial, lymphoid, and myeloid compartments may form disease-relevant microanatomical niches rather than diffuse immune-cell changes (21). This helps explain why CD163 showed a more visible signal than CD8, FOXP3, or PD-L1.

The restriction to thymoma-associated myasthenia gravis was central to the study design. Patients with sporadic thymoma were excluded because the study addressed embolized thymoma within the clinical context of MG, MG-directed therapy, autoantibody status, perioperative neurological risk, and TAMG-related immune architecture. This improves clinical focus but limits generalizability. TAMG is linked to abnormal thymic epithelial antigen presentation, altered T-cell selection, and autoimmune neuromuscular disease (22). These background immune processes complicate interpretation of post-embolization immune markers. The absence of a clear CD8, FOXP3, or PD-L1 pattern therefore does not mean that embolization has no immune effect. Rather, conventional single-marker immunohistochemistry may be too limited to separate ischemia-related tissue injury from the chronic autoimmune architecture of TAMG.

The added B-cell and germinal-center-related markers further support this cautious interpretation. CD20-positive B-cell density and CXCL13-positive area were examined as exploratory TAMG-related markers because B-cell aggregates and germinal-center-like organization are more directly related to thymic autoimmune activity than FOXP3, CD163, or PD-L1 alone. These markers did not show a graded increase with angiographic response. This result suggests that stronger devascularization was not clearly associated with greater B-cell or germinal-center-type autoimmune activity in the available tissue. However, these exploratory analyses were limited by residual tissue availability and by the fact that post-embolization resection specimens cannot reconstruct the pre-embolization immune state.

The exploratory postoperative MG outcomes also require restraint. Postoperative myasthenic crisis, respiratory deterioration, postoperative rescue intravenous immunoglobulin or plasma exchange, QMG improvement, and paired acetylcholine receptor antibody changes did not show a clear graded relationship with tumor blush reduction. QMG scores improved after surgery in available follow-up records, but this change cannot be attributed to embolization because all patients underwent thymectomy and perioperative MG management. The present data therefore do not establish embolization as a treatment for autoimmune symptoms. They show that stronger angiographic devascularization was associated with tissue necrosis and HIF-1α expression, while short-term MG outcomes remained exploratory.

Several limitations should be acknowledged. First, this was a retrospective single-center study with a limited sample size, and the findings require external validation. Second, all patients in the analytical cohort underwent embolization, so the study cannot determine whether embolization improves surgical, neurological, or oncological outcomes compared with no embolization. Third, tissue assessment was performed only after embolization and resection. Without paired pre-embolization tissue, changes in HIF-1α, VEGF-A, macrophage density, B-cell organization, or PD-L1 expression cannot be measured within the same patient. Fourth, MG-directed therapies, including corticosteroids, non-corticosteroid immunosuppressants, intravenous immunoglobulin, and plasma exchange, may have influenced immune-marker profiles despite sensitivity analyses. Fifth, single-marker immunohistochemistry cannot fully define macrophage function, T-cell state, B-cell organization, or hypoxia-related transcriptional programs. Larger prospective studies should combine standardized angiographic quantification with spatially resolved tissue analysis, multiplex immunofluorescence, transcriptomic profiling, and structured neurological follow-up (23–25).

## 5 Conclusion

In patients with thymoma-associated myasthenia gravis who underwent clinically indicated preoperative transarterial embolization, greater angiographic tumor blush reduction was associated with increased tumor necrosis proportion and higher HIF-1α expression. The HIF-1α association was attenuated after adjustment for necrosis, supporting a necrosis-linked peri-necrotic hypoxia interpretation rather than a direct causal claim. Immune findings were more heterogeneous. CD163-positive macrophage density showed an exploratory signal, whereas T-cell markers, PD-L1, the composite immune-enriched phenotype, and exploratory CD20/CXCL13 markers did not show consistent response-dependent patterns. Short-term postoperative MG outcomes also did not show a clear graded association with angiographic response. These findings support a restrained conclusion: angiographic hemodynamic response after embolization is associated mainly with ischemic and hypoxia-related pathological features in resected TAMG tissue, while autoimmune and immune-microenvironmental implications require prospective validation.

## Data Availability

The original contributions presented in the study are included in the article/**Supplementary Material**. Further inquiries can be directed to the corresponding author/s.

## References

[1] Gerber TS Strobl S Marx A Roth W Porubsky S . Epidemiology of thymomas and thymic carcinomas in the United States and Germany, 1999–2019. Front Oncol. (2024) 13:1308989. doi: 10.3389/fonc.2023.1308989 38264756 PMC10805269

[2] Marx A Chan JKC Chalabreysse L Dacic S Detterbeck F French CA . The 2021 WHO classification of tumors of the thymus and mediastinum: what is new in thymic epithelial, germ cell, and mesenchymal tumors? J Thorac Oncol. (2022) 17:200–13. doi: 10.1016/j.jtho.2021.10.010 34695605

[3] Gilhus NE . Myasthenia gravis. New Engl J Med. (2016) 375:2570–81. doi: 10.1056/NEJMra1602678 28029925

[4] Okuno T Koizumi N Yasumizu Y . Pathogenesis of thymoma-associated myasthenia gravis: a narrative review. Mediastinum. (2025) 9:26. doi: 10.21037/med-25-28 41098393 PMC12518592

[5] Lucarelli NM Maggialetti N Marulli G Mariani P Villanova I Mirabile A . Preoperative embolization in the management of giant thoracic tumors: a case series. J Pers Med. (2024) 14:1019. doi: 10.3390/jpm14101019 39452527 PMC11508663

[6] Liu K Min XL Peng J Yang K Yang L Zhang XM . The changes of HIF-1α and VEGF expression after TACE in patients with hepatocellular carcinoma. J Clin Med Res. (2016) 8:297–302. doi: 10.14740/jocmr2486w 26985249 PMC4780492

[7] Masaoutis C Palamaris K Kokkali S Levidou G Theocharis S . Unraveling the immune microenvironment of thymic epithelial tumors: implications for autoimmunity and treatment. Int J Mol Sci. (2022) 23:7864. doi: 10.3390/ijms23147864 35887212 PMC9323059

[8] Xin Z Lin M Hao Z Chen D Chen Y Chen X . The immune landscape of human thymic epithelial tumors. Nat Commun. (2022) 13:5463. doi: 10.1038/s41467-022-33170-7 36115836 PMC9482639

[9] Song JS Kim D Kwon JH Kim HR Choi CM Jang SJ . Clinicopathologic significance and immunogenomic analysis of programmed death-ligand 1 and programmed death 1 expression in thymic epithelial tumors. Front Oncol. (2019) 9:1055. doi: 10.3389/fonc.2019.01055 31681591 PMC6803548

[10] von Elm E Altman DG Egger M Pocock SJ Gøtzsche PC Vandenbroucke JP . The Strengthening the Reporting of Observational Studies in Epidemiology statement: guidelines for reporting observational studies. PloS Med. (2007) 4:e296. doi: 10.1371/journal.pmed.0040296 17941714 PMC2020495

[11] Jaretzki A 3rd Barohn RJ Ernstoff RM Kaminski HJ Keesey JC Penn AS . Myasthenia gravis: recommendations for clinical research standards. Neurology. (2000) 55:16–23. doi: 10.1212/WNL.55.1.16 10891897

[12] WHO Classification of Tumours Editorial Board . Thoracic Tumours. 5th ed. Lyon: International Agency for Research on Cancer (2021).

[13] Detterbeck FC Stratton K Giroux D Asamura H Crowley J Falkson C . The IASLC/ITMIG Thymic Epithelial Tumors Staging Project: proposal for an evidence-based stage classification system for the forthcoming eighth edition of the TNM classification of Malignant tumors. J Thorac Oncol. (2014) 9:S65–72. doi: 10.1097/JTO.0000000000000290 25396314

[14] Liu FY Wang MQ Fan QS Duan F Wang ZJ Song P . Interventional embolization of giant thoracic tumors before surgical resection. Acta Radiologica. (2013) 54:61–6. doi: 10.1258/ar.2012.120339 23377877

[15] Liu Q Fan D Adah D Wu Z Liu R Yan QT . CRISPR/Cas9-mediated hypoxia inducible factor-1α knockout enhances the antitumor effect of transarterial embolization in hepatocellular carcinoma. Oncol Rep. (2018) 40:2547–57. doi: 10.3892/or.2018.6667 30226584 PMC6151876

[16] Yan X Zhang S Deng Y Wang P Hou Q Xu H . The expression of PD-L1 and B7-H4 in thymic epithelial tumors and its relationship with tumor immune microenvironment. Front Oncol. (2021) 11:662010. doi: 10.3389/fonc.2021.662010 34307135 PMC8297388

[17] Roach C Zhang N Corigliano E Jansson M Toland G Ponto G . Development of a companion diagnostic PD-L1 immunohistochemistry assay for pembrolizumab therapy in non-small-cell lung cancer. Appl Immunohisto M M. (2016) 24:392–7. doi: 10.1097/PAI.0000000000000408 27333219 PMC4957959

[18] Khairy M Othman MH Ali EM Eldin EN . Preoperative embolization in surgical management of massive thoracic tumors. Asian Card Thorac. (2012) 20:689–93. doi: 10.1177/0218492312453462 23284111

[19] Semenza GL . HIF-1 and tumor progression: pathophysiology and therapeutics. Trends Mol Med. (2002) 8:S62–7. doi: 10.1016/S1471-4914(02)02317-1 11927290

[20] Agrafiotis AC Kokkali S Kotsantis I Theocharis S . Tumor microenvironment in thymic epithelial tumors. Cancers. (2022) 14:5905. doi: 10.3390/cancers14235905 36551568 PMC9775621

[21] Yasumizu Y Ohkura N Murata H Kinoshita M Funaki S Nojima S . Myasthenia gravis-specific aberrant neuromuscular gene expression by medullary thymic epithelial cells in thymoma. Nat Commun. (2022) 13:4230. doi: 10.1038/s41467-022-31951-8 35869073 PMC9305039

[22] Yasumizu Y Kinoshita M Zhang MJ Motooka D Suzuki K Nojima S . Spatial transcriptomics elucidates medulla niche supporting germinal center response in myasthenia gravis-associated thymoma. Cell Rep. (2024) 43:114677. doi: 10.1016/j.celrep.2024.114677 39180749

[23] Stergiou IE Palla VV Markaki S Panagiotou M Kokkali S Levidou G . PD-L1 expression in neoplastic and immune cells of thymic epithelial tumors: correlations with disease characteristics and HDAC expression. Biomedicines. (2024) 12:772. doi: 10.3390/biomedicines12040772 38672128 PMC11048374

[24] Doedens AL Stockmann C Rubinstein MP Liao D Zhang N DeNardo DG . Macrophage expression of hypoxia-inducible factor-1α suppresses T-cell function and promotes tumor progression. Cancer Res. (2010) 70:7465–75. doi: 10.1158/0008-5472.CAN-10-1439 20841473 PMC2948598

[25] Petrova V Annicchiarico-Petruzzelli M Melino G Amelio I . The hypoxic tumour microenvironment. Oncogenesis. (2018) 7:10. doi: 10.1038/s41389-017-0011-9 29362402 PMC5833859

