# Supplementary material for: Hemodynamic response after preoperative embolization in thymoma-associated myasthenia gravis: associations with necrosis, HIF-1α expression, and CD163-positive macrophages
Source: Front Immunol. 2026 Aug 18;17:1926398. doi: 10.3389/fimmu.2026.1926398 (PMC13529992; doi:10.3389/fimmu.2026.1926398)
Supplement: Supplementary file 1 [file Table1.docx]

**Supplementary Table S1.** Definitions and measurement rules for study variables.

| **Domain** | **Variable** | **Definition and measurement rule** | **Data source** | **Analytical role** |
| --- | --- | --- | --- | --- |
| Demographics | Age | Age at surgical resection, recorded in years | Medical record | Baseline covariate |
| Demographics | Sex | Male or female, recorded from the medical record | Medical record | Baseline covariate |
| Demographics | Body mass index | Weight divided by height squared, kg/m² | Medical record | Baseline covariate |
| Comorbidity | Hypertension | Documented diagnosis or use of antihypertensive medication before embolization | Medical record | Baseline covariate |
| Comorbidity | Diabetes mellitus | Documented diagnosis or use of glucose-lowering medication before embolization | Medical record | Baseline covariate |
| Comorbidity | Coronary artery disease | Documented coronary artery disease, prior myocardial infarction, prior coronary intervention, or antianginal therapy before embolization | Medical record | Baseline covariate |
| Myasthenia gravis | Disease duration | Time from first documented MG symptom to embolization, recorded in months | Neurology record | Baseline covariate |
| Myasthenia gravis | MGFA class | MGFA clinical class recorded closest to embolization and before perioperative rescue therapy when applicable | Neurology record | Sensitivity covariate |
| Myasthenia gravis | QMG score | Quantitative Myasthenia Gravis score recorded closest to embolization and before perioperative rescue therapy when applicable | Neurology record | Sensitivity covariate |
| Myasthenia gravis | AChR antibody status | Serum acetylcholine receptor antibody result, recorded as positive or negative according to the laboratory report | Laboratory record | Baseline autoimmune variable |
| Myasthenia gravis | AChR antibody level | Serum acetylcholine receptor antibody level, nmol/L, from the closest available preoperative test before embolization | Laboratory record | Baseline autoimmune variable |
| Myasthenia gravis | MuSK antibody status | Positive, negative, or not tested according to the laboratory report when tested | Laboratory record | Descriptive variable |
| MG treatment | Pyridostigmine use | Pyridostigmine use at the time of embolization, recorded as yes or no | Medical record | Baseline treatment variable |
| MG treatment | Corticosteroid use | Systemic corticosteroid use within 30 days before embolization | Medical record | Sensitivity covariate |
| MG treatment | Prednisone-equivalent dose | Daily systemic corticosteroid dose converted to prednisone-equivalent mg/day at the time closest to embolization | Medical record | Sensitivity covariate |
| MG treatment | Non-corticosteroid immunosuppressant use | Use of azathioprine, tacrolimus, cyclosporine, mycophenolate mofetil, or comparable steroid-sparing immunosuppressant for at least 4 weeks before embolization | Medical record | Sensitivity covariate |
| MG treatment | Intravenous immunoglobulin | Intravenous immunoglobulin administration within 30 days before surgery | Medical record | Sensitivity covariate |
| MG treatment | Plasma exchange | Plasma exchange performed within 30 days before surgery for MG optimization or perioperative neurological risk reduction | Neurology and procedure records | Sensitivity covariate |
| Imaging | Tumor enhancement | Mean HU from three regions of interest in enhancing solid tumor tissue on arterial-phase CT/CTA | CT/CTA | Baseline imaging variable |
| Imaging | Hypervascularity | Arterial-phase tumor enhancement greater than adjacent skeletal muscle with identifiable tumor-feeding vessels | CT/CTA | Eligibility variable |
| Embolization | Feeding arteries | Main tumor-feeding arteries identified on pre-embolization angiography | DSA report | Procedural variable |
| Embolization | Embolic material | Polyvinyl alcohol particles, gelatin sponge particles, coils, or combined use | Procedure record | Procedural variable |
| Embolization | Embolization-to-surgery interval | Time from completion of embolization to surgical incision, days | Procedure and surgical records | Core adjustment variable |
| Hemodynamic response | Angiographic tumor blush reduction | Percentage reduction in background-corrected tumor blush intensity between baseline and completion DSA | DSA images | Primary exposure |
| Hemodynamic response | Hemodynamic response group | Low response <45%, intermediate response 45%–65%, and high response >65% tumor blush reduction | DSA images | Descriptive grouping variable |
| Pathology | Tumor necrosis proportion | Necrotic thymoma area divided by total thymoma area, expressed as percentage | H&E slides | Primary pathological outcome |
| Hypoxia-related marker | HIF-1α H-score | Tumor-cell H-score from 0 to 300, prioritizing nuclear staining | IHC slide | Primary molecular outcome |
| Hypoxia-related marker | CAIX H-score | Tumor-cell H-score from 0 to 300 based on membranous or membranous-cytoplasmic staining | IHC slide | Secondary molecular outcome |
| Hypoxia-related marker | VEGF-A H-score | Tumor-cell H-score from 0 to 300 based on cytoplasmic staining | IHC slide | Secondary molecular outcome |
| Immune microenvironment | CD8-positive T-cell density | CD8-positive cells per mm² of viable tumor area | IHC slide | Secondary immune outcome |
| Immune microenvironment | FOXP3-positive T-cell density | FOXP3-positive cells per mm² of viable tumor area | IHC slide | Secondary immune outcome |
| Immune microenvironment | CD163-positive macrophage density | CD163-positive cells per mm² of viable tumor area | IHC slide | Primary immune outcome |
| Immune microenvironment | PD-L1 TPS | Percentage of viable tumor cells with partial or complete membranous PD-L1 staining | IHC slide | Secondary immune outcome |
| Immune microenvironment | CD8/FOXP3 ratio | CD8-positive T-cell density divided by FOXP3-positive T-cell density; cases with FOXP3 density of 0 were treated as missing for this ratio | IHC slide | Secondary immune outcome |
| Composite endpoint | Composite immune-enriched phenotype | Presence of at least two of the following: FOXP3-positive T-cell density above the cohort median, CD163-positive macrophage density above the cohort median, and PD-L1 TPS above the cohort median | IHC slide | Secondary exploratory binary outcome |
| Surgical outcome | Operative approach | Video-assisted thoracoscopic surgery, robotic-assisted thoracic surgery, sternotomy, thoracotomy, or combined approach | Surgical record | Perioperative variable |
| Surgical outcome | Completeness of resection | R0, R1, or R2 according to operative and pathological reports | Surgical and pathology records | Perioperative variable |
| Surgical outcome | Intraoperative blood loss | Estimated blood loss during surgery, mL | Anesthesia record | Perioperative outcome |
| Safety outcome | Embolization-related adverse event | Chest pain, fever, access-site hematoma, non-target embolization, respiratory deterioration, or transient MG worsening within 72 hours after embolization | Procedure and ward records | Safety outcome |
| Safety outcome | Postoperative myasthenic crisis | Postoperative respiratory failure or severe MG deterioration requiring prolonged mechanical ventilation, reintubation, rescue ventilation, or urgent ventilatory support during the index hospitalization and within 30 days after surgery | Medical record | Safety outcome; exploratory MG-related outcome |
| Neurological outcome | Postoperative QMG reassessment | First available QMG score after surgery, preferably before discharge or at the first postoperative neurology follow-up | Neurology record | Exploratory MG-related outcome |
| Neurological outcome | Three-month QMG score | QMG score recorded at the follow-up visit closest to 3 months after surgery when available | Neurology record | Exploratory MG-related outcome |
| Autoimmune outcome | Postoperative AChR antibody level | Serum acetylcholine receptor antibody level from the closest available postoperative follow-up when available | Laboratory record | Exploratory autoimmune outcome |
| Hospital course | Intensive-care-unit admission | ICU admission after surgery, recorded as yes or no | Medical record | Perioperative outcome |
| Hospital course | Postoperative hospital stay | Days from surgery to discharge | Medical record | Perioperative outcome |

Abbreviations: AChR, acetylcholine receptor; CAIX, carbonic anhydrase IX; CT, computed tomography; CTA, computed tomography angiography; DSA, digital subtraction angiography; H&E, hematoxylin and eosin; HIF-1α, hypoxia-inducible factor-1α; HU, Hounsfield unit; ICU, intensive-care unit; IHC, immunohistochemistry; MG, myasthenia gravis; MGFA, Myasthenia Gravis Foundation of America; MuSK, muscle-specific kinase; PD-L1, programmed death-ligand 1; QMG, Quantitative Myasthenia Gravis; TPS, tumor proportion score; VEGF-A, vascular endothelial growth factor A; WHO, World Health Organization.

Supplementary Table S2. MG-directed therapy variables and exploratory MG outcome definitions.

| **Domain** | **Variable** | **Definition and measurement rule** | **Measurement window** | **Coding or unit** | **Data source** | **Analytical role** |
| --- | --- | --- | --- | --- | --- | --- |
| Baseline MG severity | MGFA class | MGFA clinical class assigned by the treating neurologist | Closest assessment before embolization and before perioperative rescue therapy | I, II, III, IV, or V | Neurology record | Baseline MG severity variable; sensitivity covariate |
| Baseline MG severity | QMG score | Quantitative Myasthenia Gravis score recorded by the neurology team | Closest assessment before embolization and before perioperative rescue therapy | Points | Neurology record | Baseline MG severity variable; sensitivity covariate |
| Autoimmune status | AChR antibody status | Serum acetylcholine receptor antibody result | Closest available preoperative test before embolization | Positive or negative | Laboratory record | Baseline autoimmune variable |
| Autoimmune status | AChR antibody level | Quantitative serum acetylcholine receptor antibody concentration | Closest available preoperative test before embolization | nmol/L | Laboratory record | Baseline autoimmune variable; paired exploratory outcome when postoperative value was available |
| Autoimmune status | MuSK antibody status | Muscle-specific kinase antibody result when tested | Closest available preoperative test before embolization | Positive, negative, or not tested | Laboratory record | Baseline autoimmune variable |
| Symptomatic MG treatment | Pyridostigmine use | Documented pyridostigmine use at the time of embolization | Medication record closest to embolization | Yes or no | Medical record | Baseline treatment variable |
| Corticosteroid therapy | Corticosteroid exposure | Any systemic corticosteroid treatment before embolization | Within 30 days before embolization | Yes or no | Medical record | Potential confounder for immune-marker analyses |
| Corticosteroid therapy | Prednisone-equivalent dose | Daily systemic corticosteroid dose converted to prednisone-equivalent dose | Dose closest to embolization | mg/day | Medical record | Sensitivity covariate for immune-marker analyses |
| Non-corticosteroid immunosuppression | Non-corticosteroid immunosuppressant use | Use of azathioprine, tacrolimus, cyclosporine, mycophenolate mofetil, or comparable steroid-sparing immunosuppressant | Active use for at least 4 weeks before embolization | Yes or no | Medical record | Potential confounder for immune-marker analyses |
| Perioperative immune therapy | Intravenous immunoglobulin | Intravenous immunoglobulin administered for MG optimization or perioperative neurological risk reduction | Within 30 days before surgery | Yes or no | Neurology and medication records | Potential confounder for immune-marker and perioperative MG outcome analyses |
| Perioperative immune therapy | Plasma exchange | Plasma exchange performed for MG optimization or perioperative neurological risk reduction | Within 30 days before surgery | Yes or no | Neurology and procedure records | Potential confounder for immune-marker and perioperative MG outcome analyses |
| Early postoperative MG outcome | Postoperative myasthenic crisis | Postoperative respiratory failure or severe MG deterioration requiring prolonged mechanical ventilation, reintubation, rescue ventilation, or urgent ventilatory support and documented as MG-related by the clinical team | During the index hospitalization and within 30 days after surgery | Yes or no | Surgical, ICU, anesthesia, and neurology records | Exploratory MG-related outcome |
| Early postoperative respiratory outcome | Respiratory deterioration | New or worsened postoperative respiratory weakness requiring non-invasive ventilation, invasive ventilation, ICU-level respiratory monitoring, or urgent neurological reassessment | During the index hospitalization | Yes or no | ICU, anesthesia, surgical, and neurology records | Exploratory MG-related outcome |
| Early postoperative MG therapy | Postoperative intravenous immunoglobulin or plasma exchange | Escalation to intravenous immunoglobulin or plasma exchange after surgery because of MG worsening, myasthenic crisis, or high-risk neurological status | During the index hospitalization | Yes or no | Neurology, medication, and procedure records | Exploratory MG-related outcome |
| Short-term neurological status | First postoperative QMG score | First QMG score recorded after surgery | Preferably before discharge; otherwise first available postoperative neurology follow-up | Points | Neurology record | Exploratory MG-related outcome |
| Short-term neurological change | Early QMG change | Difference between first postoperative QMG score and baseline QMG score | Baseline to first postoperative reassessment | Points; postoperative QMG minus baseline QMG | Neurology record | Exploratory MG-related outcome |
| Three-month neurological status | Three-month QMG score | QMG score recorded at short-term postoperative follow-up | Closest available assessment to 3 months after surgery | Points | Neurology record | Exploratory MG-related outcome |
| Three-month neurological change | Three-month QMG improvement | Change in QMG score from baseline to approximately 3 months after surgery | Baseline to closest available 3-month postoperative reassessment | Points; baseline QMG minus 3-month QMG | Neurology record | Exploratory MG-related outcome |
| Short-term autoimmune change | Postoperative AChR antibody level | Quantitative serum acetylcholine receptor antibody concentration after surgery when available | Closest available postoperative follow-up | nmol/L | Laboratory record | Exploratory autoimmune outcome |
| Short-term autoimmune change | Change in AChR antibody level | Difference between postoperative and preoperative AChR antibody levels when paired values were available | Baseline to closest available postoperative follow-up | nmol/L; postoperative value minus baseline value | Laboratory record | Exploratory autoimmune outcome |
| Postoperative care intensity | ICU admission | Admission to the intensive-care unit after surgery for planned monitoring, respiratory support, myasthenic crisis, or other perioperative indications | During the index postoperative hospitalization | Yes or no | Surgical and ICU records | Perioperative outcome; exploratory care-intensity marker |
| Postoperative recovery | Postoperative hospital stay | Number of days from surgery to discharge | Index hospitalization | Days | Discharge record | Perioperative outcome |

Abbreviations: AChR, acetylcholine receptor; ICU, intensive-care unit; MG, myasthenia gravis; MGFA, Myasthenia Gravis Foundation of America; MuSK, muscle-specific kinase; QMG, Quantitative Myasthenia Gravis.
